# Risk Factors for Hepatitis C Virus Among the General Population in Sub‐Saharan Africa—An Analysis of Systematic Review Data

**DOI:** 10.1111/jvh.70065

**Published:** 2025-09-01

**Authors:** Getahun Molla Kassa, Aaron G. Lim, Melaku Tileku Tamiru, Tesfa Sewunet Alamneh, Peter Vickerman, Emebet Dagne, Andargachew Mulu, Obsie Baissa, Ora Paltiel, John F. Dillon, Elias Ali Yesuf, Matthew Hickman, Josephine G. Walker, Clare E. French, DESTINE NIHR Global Health Research Group, Wondwossen Amogne, Dawit Wolday, Aynishet Adane, Saro Abdella, Zenahbezu Abay, Workagegnehu Hailu, Tadesse Awoke, Atsbeha Gebreegziabxier Weldemariam, Christie Cabral, Elizabeth Speakman, Andrew Radley, Amy Malaguti, Sarah Inglis, Meseret Yohannes, Bruktait Taddele, Hagos Abraha, Mengistu Erkie

**Affiliations:** ^1^ Population Health Sciences, Bristol Medical School University of Bristol Bristol UK; ^2^ Department of Epidemiology and Biostatistics, Institute of Public Health, College of Medicine and Health Sciences University of Gondar Gondar Ethiopia; ^3^ Division of Molecular and Clinical Medicine, School of Medicine University of Dundee Dundee UK; ^4^ Department of Pharmacology and Clinical Pharmacy, School of Pharmacy, College of Health Sciences Addis Ababa University Addis Ababa Ethiopia; ^5^ Department of Internal Medicine, Institute of Health Jimma University Jimma Ethiopia; ^6^ Armauer Hansen Research Institute Addis Ababa Ethiopia; ^7^ Department of Hematology and Braun School of Public Health, Faculty of Medicine Hadassah‐Hebrew University Jerusalem Israel; ^8^ Department of Health Policy and Management, Institute of Health Jimma University Jimma Ethiopia; ^9^ National Drug and Alcohol Research Centre University of New South Wales Sydney Australia; ^10^ NIHR Health Protection Research Unit in Behavioural Science and Evaluation University of Bristol Bristol UK

**Keywords:** general population, hepatitis C, meta‐analysis, risk factors, sub‐Saharan Africa

## Abstract

Understanding risk factors for hepatitis C virus (HCV) is critical for targeting screening and prevention. We systematically reviewed risk factors associated with HCV seroprevalence among the general population in sub‐Saharan Africa (SSA). Comprehensive systematic review of HCV seroprevalence of community‐based observational studies reporting HCV risk factors in SSA. Study quality was assessed using Joanna Briggs Institute tool. Random effect meta‐analyses were used to estimate odds ratios (OR) with 95% confidence intervals (CI). We identified 92 studies. Higher odds of HCV seroprevalence were observed among age 21–64 (OR = 1.77, 95% CI 1.17–2.68) and 65+ groups (OR = 11.75, 95% CI 5.51–25.05) compared to those aged ≤ 20 years; not being formally educated (OR = 1.78, 95% CI 1.35–2.35) compared to secondary/above and being married (OR = 1.91, 95% CI 1.45–2.51) or divorced (OR = 3.20, 95% CI 1.91–5.36) compared to never married. Family history of HCV (OR = 1.52, 95% CI 1.17–1.96), being a person living with HIV (OR = 2.64, 95% CI 1.61–4.33) or being HBsAg positive (OR = 1.66, 95% CI 1.10–2.50) were all positively associated with increased HCV seroprevalence, as was having a history of blood transfusion (OR = 1.81, 95% CI 1.33–2.45), hospitalisation (OR = 1.55, 95% CI 1.22–1.96), medical operation (OR = 1.28, 95% CI 1.01–1.62), scarification (OR = 1.29, 95% CI 1.01–1.64) and injection drug use (OR = 7.04, 95% CI 1.16–42.68). Pilot HCV screening programmes targeting older adults and people exposed to healthcare‐associated factors could potentially lead to the efficient detection of HCV cases and reduce future HCV exposures among the general population in SSA countries.

**Abbreviations**
%OR: OR change
CI: confidence interval
DAAs: direct‐acting antivirals
ELISA: enzyme‐linked immunosorbent assay
HBsAg: hepatitis B virus surface antigen
HCV: hepatic C virus
I^2^
: heterogeneity statistics and percent
IDU: injection drug use
JBI: Joanna Briggs Institute
MSM: men who have sex with men
OR: odds ratio
PLWH: people living with HIV
PRISMA: Preferred Reporting Items for Systematic Review and Meta‐Analysis
PWID: people who inject drugs
RDT: rapid diagnostic test kits
RR: relative risk
SSA: sub‐Saharan Africa
WHO: World Health Organization

## 1 Introduction

Hepatitis C Virus (HCV) is one of the leading causes of liver‐related morbidity and mortality worldwide [1]. In 2022, 50 million people had chronic HCV infection globally, with 7.8 million in the World Health Organization (WHO) African region [2].

It is hypothesised that the predominant route for HCV infection in sub‐Saharan Africa (SSA) is through unsafe medical practices [3]. Globally, people who inject drugs (PWID), people living with HIV (PLWH), people who have had medical injections and men who have sex with men are high‐risk populations for HCV [4, 5]. However, evidence on these risk factors is scarce from low‐ to middle‐income countries and SSA.

HCV transmission can be reduced through a combination of interventions. These include expanding access to HCV screening and treatment and reducing the risk of exposure through implementing preventive programs such as health education in diverse settings to increase public awareness [4, 6]. The availability of preventive programs and effective treatment has substantially decreased the incidence of HCV in some countries, particularly following the introduction of highly effective direct‐acting antivirals (DAAs) that are associated with a 95% cure rate, leading to reduced liver disease progression and hepatitis‐related death and preventing secondary transmission [4, 7].

In the era of DAAs, universal HCV screening of the entire general population appears cost‐effective in some settings [8, 9], but it might not be feasible or affordable in SSA. Most SSA countries remain unable to add DAAs to their national essential drugs lists [10, 11]. Therefore, selective/targeted screening based on exposure to risk factors or individuals from high prevalence populations, with integration into existing healthcare services such as antenatal care or HIV clinics has been recommended [3, 12]. Here, we investigate and quantify risk factors associated with HCV seroprevalence among the general population in SSA to guide prevention activities, help inform tailored screening strategies for community‐based case finding, prioritise population groups for screening and improve HCV surveillance.

## 2 Methods

### 2.1 Study Design

We meta‐analysed data from studies identified through a previously published systematic review on the prevalence of HCV in SSA [13], with additional data on risk factors extracted from the included studies for this analysis. We report methods and results according to the Preferred Reporting Items for Systematic Review and Meta‐Analysis (PRISMA) 2020 guidelines [14]. The systematic review protocol is registered in PROSPERO (CRD42023373986): https://www.crd.york.ac.uk/PROSPERO/view/CRD42023373986.

### 2.2 Search Strategy

In summary, for the systematic review, we comprehensively searched Ovid Medline, Embase, APA PsycINFO, Web of Science and WHO African Medicos Index to December 20th, 2023, for papers that reported HCV prevalence within the general population in SSA. See Table S1 for search strategy; full details are provided within the published main review [13].

### 2.3 Eligibility and Selection Criteria

Titles/abstracts and full texts were double screened independently. Detailed inclusion and exclusion criteria are available within the published review [13]. For this analysis, from the studies included in the published review, we included community‐based cohort, cross‐sectional and case–control studies that were conducted among general population samples and reported at least one sociodemographic, behavioural, social, medical or other risk factors directly or indirectly associated with HCV antibody positivity [13].

### 2.4 Quality Assessment

We used the Joanna Briggs Institute (JBI) quality assessment tool to assess the quality of included studies [15]. Detailed assessment criteria are reported in the published review [13].

### 2.5 Data Extraction

Data were extracted by two independent reviewers in Covidence [16], and discrepancies were managed by discussion. We performed an initial review of all eligible studies and specified risk factors including age, sex, marital status, educational status, residency (urban/rural), occupation, HIV, family history of HCV, blood transfusion, medical injections, hospitalisation, scarification/tattooing, dental procedures, injured by or shared sharp materials, history of injection drug use (IDU), history of multiple sexual partners, bought/sold sex, male circumcision, female genital mutilation and place of male circumcision or female genital mutilation. All were extracted from each study as follows, with additional risk factors identified through the data extraction process: we extracted summary frequencies of HCV positive and negatives along with associated crude and adjusted odds ratios (OR) or relative risks (RR) and 95% confidence intervals (CI) if available. Related/similar risk factors were grouped together (e.g., blood and blood product transfusions were combined into ‘blood transfusions’; Table S3). Many studies were conducted for two age groups (≤ 20 and 21–64 years), whereas others included all age groups. Similarly, some studies only compared never married/single versus married while some others also compared never married/single versus divorced. Therefore, the number of studies comparing age ≤ 20 versus 21–64 differed from those comparing age ≤ 20 versus 65+; as well as never married/single vs. married and never married/single vs. divorced.

### 2.6 Ascertainment of HCV Antibody Positivity

We included studies that used either enzyme‐linked immunosorbent assay (ELISA) or rapid diagnostic test kits (RDT) for detecting anti‐HCV antibody. Where studies used multiple test methods, such as sequential or parallel tests, we took results reported from the confirmatory HCV antibody test.

### 2.7 Data Analyses

Study characteristics were summarised according to study design, study period, geographical location and HCV testing methods. All reported risk factors were listed in a table, and those risk factors reported from five or more studies were considered in the meta‐analyses. However, different occupations and wealth indices were reported in the included studies, which prevented meta‐analysis since it was impossible to create a common categorisation for these variables across the included studies. Reported frequencies for HCV positive and negative (crude) across each variable category were used to estimate the summary effect size (OR) since limited studies reported adjusted OR for only a few variables, and the adjustment variables widely varied across studies.

Random effect meta‐analyses were run using the *meta esize* command in Stata/MP 18 [17] for each risk factor separately to estimate the summary effect size (OR with its 95% CI and prediction interval (PI)). We applied the restricted maximum likelihood (REML) method to provide precise variance estimation. Zero observed cells were adjusted using the value of 0.5. Heterogeneity was assessed using the index of heterogeneity statistics (*I* ^2^) and *Q* test. Effect estimates were visualised using forest plots.

Subgroup analyses were performed to assess whether risk factors varied by SSA subregion. We were unable to analyse any risk factors for the Southern Africa region due to few studies (*n* = 2).

Three sensitivity analyses were run. First, restricting to high‐quality studies. Second, we planned to run a subgroup analysis by age group (≤ 20, 21–64 and 65+) to minimise the confounding effect of age; however, there were very few studies conducted only for age ≤ 20 and 65+. Therefore, we instead ran a sensitivity analysis restricted to studies that were conducted across all age groups, which might help to adjust for the effect of age. Third, we restricted to studies conducted in the most recent time period (since 2015) as recent improvements in the safety of healthcare practices may have reduced the risks of healthcare‐associated factors [18, 19].

We calculated OR percent change (%OR) for each significantly associated risk factor in the main meta‐analysis and sensitivity analyses using the formula described below. OR1 is OR estimated from the main meta‐analysis and OR2 is OR estimated from the sensitivity analysis.

$$\%\text{OR}=\left|\text{OR}1-\text{OR}2\right|*100/\text{OR}1$$

## 3 Results

### 3.1 Characteristics of Included Studies

Our systematic review on the prevalence of HCV in SSA [13] identified 130 eligible studies, of which 92 reported at least one risk factor for HCV and were thus included in this analysis. There was a total of 1,860,611 study subjects. Studies were from 25/51 SSA countries (which represented 73.8% of SSA population), predominantly from Nigeria (*n* = 22/92, 23.9%), Cameroon (*n* = 15/92, 16.3%) and Ethiopia (*n* = 11/92, 12.0%; Figure S1). Eastern Africa (*n* = 35, 38.0%) and Western Africa (*n* = 32, 34.8%) contributed the largest number followed by Central Africa (*n* = 23, 25.0%) and Southern Africa (*n* = 2, 2.2%), respectively. All were cross‐sectional studies. The study participants were either a mixed group (all ages and both sexes; *n* = 53, 57.6%), adults (male and female; *n* = 13, 4.1%), students (*n* = 10, 10.8%), children (*n* = 7, 7.6%), adult women (*n* = 5, 5.4%), older populations (> 50 years; *n* = 3, 3.3%) and adult males (*n* = 1, 1.1%). Nearly, three quarters (*n* = 68, 73.9%) used ELISA, 20.7% (*n* = 19) used RDT, and 5.4% (*n* = 5) used unspecified testing methods to diagnose HCV antibody. The number of studies was evenly distributed across the three time periods: 30.4% (*n* = 28) for 1984–2000, 38.0% (*n* = 35) for 2001–2014 and 31.5% (*n* = 29) 2015–2023. In the JBI quality assessment, 50.0% (*n* = 46) of studies scored high quality, 28.3% (*n* = 26) moderate quality and 21.7% (*n* = 20) poor quality (Figure S2). Detailed characteristics of the included studies are in Table S8.

### 3.2 Risk Factors Included in the Analysis

The complete list of all risk factors reported from the included studies is in Table S2. In total, 88 studies contributed to the meta‐analyses—Table 1 shows risk factors that were reported from ≥ 5 studies and contributed to the meta‐analyses.

**TABLE 1:** Characteristics of the risk factors included in the meta‐analysis model.

Risk factors	No. of studies	Country from (no. of studies)	Study population (no. of studies)	Study period (no. of studies)
Gender/sex	74	Burkina Faso (2), Benin (1), Burundi (1), Cameroon (13), Central Africa Republic (2), DR Congo (1), Equatorial Guinea (1), Ethiopia (6), Gabon (3), Ghana (1), Guinea Bissau (2), Kenya (2), Madagascar (2), Mayotte (1), Nigeria (19), Rwanda (5), Seychelles (1), Sierra Leone (1), South Africa (1), Sudan (2), Tanzania (5), Zimbabwe (1) and Republic of Congo (1)	Adult (10), children (6), Mixed group (45), Old (3) and Student (10)	1984–2000 (20), 2001–2014 (31) and 2015–2023 (23)
Age in years	40	Burkina Faso (2), Benin (1), Burundi (1), Cameroon (5), Central Africa Republic (2), Equatorial Guinea (1), Ethiopia (5), Gabon (3), Kenya (2), Madagascar (2), Nigeria (9), Rwanda (2), Sudan (1) and Tanzania (4)	Adult (5), adult women (1), children (1), Mixed group (28) and Student (5)	1984–2000 (9), 2001–2014 (21) and 2015–2023 (10)
Educational status	13	Benin (1), Burkina Faso (2), Cameroon (1), Central Africa Republic (1), Ethiopia (5), Kenya (1), Nigeria (1) and Rwanda (1)	Adult (3), Women (2), Children (1), Mixed (6) and Old (1)	1984–2000 (0), 2001–2014 (5) and 2015–2023 (8)
Marital status	20	Benin (1), Burkina Faso (1), Cameroon (3), Ethiopia (5), Guinea Bissau (1), Kenya (2), Madagascar (1), Nigeria (3) and Rwanda (3)	Adult (5), women (2), Mixed (11), old (1) and Student (1)	1984–2000 (0), 2001–2014 (9) and 2015–2023 (11)
Urban/rural residency	12	Burkina Faso (2), Burundi (1), Cameroon (1), Central Africa Republic (1), Ethiopia (3), Kenya (1), Rwanda (1), South Africa (1) and Tanzania (1)	Adult (5) and Mixed group (7)	1984–2000 (3), 2001–2014 (5) and 2015–2023 (4)
HIV/AIDS status	19	Cameroon (2), Ethiopia (3), Guinea Bissau (2), Kenya (1), Nigeria (3), Rwanda (4), Tanzania (3) and Zimbabwe (1)	Adult (4), women (1), Children (1), Mixed (10), Old (1) and Student (1)	1984–2000 (3), 2001–2014 (6) and 2015–2023 (10)
HBsAg status	30	Benin (1), Burkina Faso (3), Cameroon (3), Central Africa Republic (1), Ethiopia (4), Guinea (1), Kenya (1), Mayotte (1), Nigeria (9), Rwanda (4), Sudan (1) and Tanzania (1)	Adult (3), Male (1), women (1), Children (2), mixed (20) and student (3)	1984–2000 (4), 2001–2014 (10) and 2015–2023 (16)
History of multiple sexual partners	14	Ethiopia (3), Kenya (1), Nigeria (7) and Rwanda (3)	Adult (1), Women (2), mixed (7) and student (4)	1984–2000 (0), 2001–2014 (4) and 2015–2023 (10)
Male circumcisions	7	Cameroon (2), DR Congo (1), Ethiopia (1), Guinea Bissau (1), Madagascar (1) and Nigeria (1)	Adult (1), Mixed (3), Old (2) and Student (1)	1984–2000 (0), 2001–2014 (5) and 2015–2023 (2)
History of medical injections	9	Cameroon (1), DR Congo (1), Ethiopia (1), Gabon (1), Kenya (1), Madagascar (1), Nigeria (2) and Sudan (1)	Adult (1), Mixed (5), Old (2) and Student (1)	1984–2000 (1), 2001–2014 (6) and 2015–2023 (2)
History of blood or blood product transfused	25	Cameroon (1), DR Congo (1), Ethiopia (3), Gabon (1), Guinea Bissau (2), Kenya (1), Madagascar (2), Nigeria (7), Rwanda (4), Sudan (1), Tanzania (1) and Zimbabwe (1)	Adult (2), Women (2), Mixed (14), Old (3) and student (4)	1984–2000 (3), 2001–2014 (10) and 2015–2023 (12)
History of injection drug use	5	Cameroon (1), Ethiopia (1), Madagascar (1) and Nigeria (2)	Mixed (4) and Student (1)	1984–2000 (0), 2001–2014 (2) and 2015–2023 (3)
History of dental extraction	5	Ethiopia (2), Madagascar (1), Rwanda (1) and Sudan (1)	Mixed (5)	1984–2000 (1), 2001–2014 (1) and 2015–2023 (3)
Family history of HCV	7	Ethiopia (2), Nigeria (1) and Rwanda (4)	Mixed (6) and women (1)	1984–2000 (0), 2001–2014 (0) and 2015–2023 (7)
History of hospitalisation	6	Ethiopia (3), Gabon (1), Madagascar (1) and Rwanda (1)	Women (1) and Mixed (5)	1984–2000 (0), 2001–2014 (2) and 2015–2023 (4)
History of medical operation/procedures	13	Cameroon (1), Ethiopia (3), Madagascar (1), Nigeria (2), Rwanda (4), Sudan (1) and Tanzania (1)	Adult (1), women (2), Mixed (9) and Student (1)	1984–2000 (2), 2001–2014 (2) and 2015–2023 (9)
Scarification/Tattooing	18	Cameroon (1), Ethiopia (4), Gabon (1), Guinea Bissau (1), Madagascar (1), Nigeria (7), Rwanda (2) and Sudan (1)	Adult (1), Women (1), Mixed (11), Old (2) and Student (3)	1984–2000 (2), 2001–2014 (8) and 2015–2023 (8)
Shared/injured by sharps/piercing materials	5	Ethiopia (1), Nigeria (3) and Rwanda (1)	Mixed (4) and Student (1)	1984–2000 (0), 2001–2014 (1) and 2015–2023 (4)
Alcohol use	7	Benin (1), Ethiopia (2), Nigeria (3) and Sudan (1)	Women (1), Mixed (4) and student (2)	1984–2000 (1), 2001–2014 (2) and 2015–2023 (4)

### 3.3 Factors Associated With HCV Seroprevalence

Table 2 shows the meta‐analysis results with summary data on HCV positive and sample size. The odds ratios of HCV infection were 1.77 (95% CI 1.17–2.68) and 11.75 (95% CI 5.52–25.05) in the age groups 21–64 and 65+ years compared to the age group ≤ 20 years, respectively. Higher odds of HCV seroprevalence were observed among those not being formally educated (OR = 1.78, 95% CI 1.35–2.35) compared to those who attended secondary/higher school and those married (OR = 1.91, 95% CI 1.45–2.51) and divorced (OR = 3.20, 95% CI 1.91–5.36) compared to those who were single/never married. PLWH (OR = 2.78, 95% CI 1.70–4.55), HBsAg positives (OR = 1.66, 95% CI 1.10–2.50) and those having a family history of HCV (OR = 1.52, 95% CI 1.17–1.96) had higher odds of HCV seroprevalence compared to their counterparts. Similarly, the odds of higher HCV seroprevalence were positively associated with blood transfusion (OR = 1.81, 95% CI 1.33–2.45), history of hospitalisation (OR = 1.55, 95% CI 1.22–1.96), having had a medical operation (OR = 1.28, 95% CI 1.01–1.62), history of IDU (OR = 7.04, 95% CI 1.16–42.68) and having scarification (OR = 1.29, 95% CI 1.01–1.64). All other risk factors had an OR of > 1, but with weak evidence as the 95% CIs cross the null. These pooled ORs and 95% CIs represent average associations which may not apply universally (e.g., in all settings/population groups). We additionally present 95% prediction intervals to show the range within which results of a new study could fall. All forest plots for each significantly associated risk factors are available in the Figures S3–S15.

**TABLE 2:** Association between risk factors and HCV seroprevalence among the general population in SSA.

Risk factors	Categories	HCV% (HCV+/Sample Size)	OR (95% CI)	OR (95% PI)	*n*, *I* ^2^
Gender/sex	Female	5.8 (59,225/1,019,239)	Reference		
	Male	5.9 (29,580/502,303)	1.01 (0.94–1.08)	1.01 (0.79–1.08)	74, 68.63
Age in years	≤ 20^a^	1.0 (677/70,150)	Reference to age 21–64		
		0.9 (458/53,376)	Reference to age 65+		
	21–64	2.5 (5645/226,458)	1.77 (1.17–2.68)*	1.77 (0.18–2.68)	40, 93.04
	65+	16.2 (9994/61,786)	11.75 (5.52–25.05)*	11.75 (0.52–266.06)	19, 96.21
Education	Not formally educated	5.0 (691/13,882)	1.78 (1.35–2.35)*	1.78 (1.03–3.08)	13, 19.66
	Primary	6.1 (449/7349)	1.29 (0.59–2.83)	1.29 (0.08–20.87)	13, 89.43
	Secondary or above	3.4 (215/6342)	Reference		
Marital status	Never married^b^	1.6 (3128/201,318)	Reference to married		
		1.6 (3088/198,706)	Reference to divorced		
	Married	4.8 (39,356/827,009)	1.91 (1.45–2.51)*	1.91 (0.73–5.01)	20, 94.35
	Divorced	7.4 (15,963/215,700)	3.20 (1.91–5.36)*	3.20 (0.58–17.36)	15, 98.18
Residency	Urban	1.9 (1741/94,082)	Reference		
	Rural	2.5 (2734/108,615)	1.32 (0.90–1.96)	1.32 (0.37–4.69)	12, 91.35
HIV	Negative	4.8 (56,755/1,180,262)	Reference		
	Positive	7.8 (2577/32,883)	2.78 (1.70–4.55)*	2.78 (0.44–17.59)	19, 97.62
HBsAg	Negative	5.0 (57,415/1,140,550)	Reference		
	Positive	3.7 (1382/37,072)	1.66 (1.10–2.50)*	1.66 (0.28–9.96)	30, 93.53
History of multiple sexual partners	No	5.3 (24,912/472,814)	Reference		
	Yes	4.6 (903/19,619)	1.24 (0.93–1.67)	1.24 (0.53–2.93)	14, 79.37
Male circumcisions	No	0.3 (1/299)	Reference		
	Yes	3.2 (215/6772)	1.36 (0.43–4.23)	1.36 (0.31–6.02)	7, 0.00
History of medical injections	No	8.7 (504/5774)	Reference		
	Yes	12.5 (644/5170)	1.27 (0.93–1.72)	1.27 (0.57–2.83)	9, 57.00
Blood transfused	No	5.0 (58,907/1,179,286)	Reference		
	Yes	5.9 (1765/29,995)	1.81 (1.33–2.45)*	1.81 (0.57–5.78)	25, 91.33
History of injection drug use	No	1.1 (80/7091)	Reference		
	Yes	1.6 (5/316)	7.04 (1.16–42.68)*	7.04 (0.02–2757.74)	5, 70.64
History of dental extraction	No	1.6 (30/1894)	Reference		
	Yes	2.6 (53/2059)	1.91 (0.77–4.74)	1.91 (0.13–27.92)	5, 52.16
Family history of HCV	No	4.3 (56,903/1,310,170)	Reference		
	Yes	6.2 (2207/35,799)	1.52 (1.17–1.96)*	1.52 (0.76–3.04)	7, 89.27
History of hospitalisation	No	4.3 (195/4513)	Reference		
	Yes	9.7 (333/3443)	1.55 (1.22–1.96)*	1.55 (1.02–2.35)	6, 4.29
History of medical operation/procedures	No	5.0 (56,092/1,131,300)	Reference		
	Yes	5.4 (3403/63,267)	1.28 (1.01–1.62)*	1.28 (0.72–2.26)	13, 87.94
History of Scarification/Tattooing	No	5.0 (21,453/430,045)	Reference		
	Yes	6.7 (5195/77,801)	1.29 (1.01–1.64)*	1.29 (0.59–2.83)	18, 89.49
History of injury by sharp materials or shared sharp materials	No	3.2 (141/4347)	Reference		
	Yes	3.5 (76/2172)	1.57 (0.77–3.20)	1.57 (0.18–13.73)	5, 64.77
Alcohol use	No	1.4 (64/4670)	Reference		
	Yes	4.7 (41/872)	2.25 (0.83–6.08)	2.25 (0.10–50.19)	7, 76.84

Abbreviations: CI, confidence interval; HBsAg, hepatitis B virus surface antigen; HCV%, HCV+/sample size; HCV, hepatitis C virus; HCV+, hepatitis C virus antibody positive; HIV, human immunodeficiency virus; *I* ^2^, index of heterogeneity statistics; *n*, number of studies; OR, odds ratio; PI, prediction intervals; sample size, HCV antibody positive plus HCV antibody negative. *significant associated factors.

^a^ Some studies have data only for age ≤ 20 and 21–64 to compare and others for age 65+ to compare to age ≤ 20 because there were some studies conducted only among those aged < 65 years and others conducted in all age groups.

^b^ Some studies only compare never married/single and married while others have also for divorced.

### 3.4 Subgroup Analysis for SSA Subregions

We meta‐analysed risk factors that were reported in ≥ 5 studies in each SSA subregion. In Central Africa, HCV seroprevalence was positively associated with age 21–64 years (OR = 5.33, 95% CI 2.90–9.79) and 65+ years (OR = 25.23, 95% CI 12.76–49.89). While in Eastern Africa, it was associated with age group 65+ (OR = 6.61, 95% CI 1.81–24.12), being married (OR = 1.84, 95% CI 1.23–2.75) and divorced (OR = 2.78, 95% CI 1.36–5.70), PLWH (OR = 2.92, 95% CI 1.76–4.84), history of blood transfusion (OR = 2.01, 95% CI 1.18–3.43), hospitalisation history (OR = 2.06, 95% CI 1.26–3.37) and having a family history of HCV (OR = 1.52, 95% CI 1.14–2.03). In Western Africa, married individuals (OR = 2.27, 95% CI 1.32–3.91), those who were HBsAg positive (OR = 1.99, 95% CI 1.08–3.67) and having a history of blood transfusion (OR = 2.05, 95% CI 1.15–3.65) had higher odds of HCV seroprevalence. Details are in Table S4.

### 3.5 Sensitivity Analyses

In all sensitivity analyses, the direction of all ORs was unchanged, but the %OR changed by more than 50% for some risk factors.

When restricted to high‐quality studies (*n* = 46), all risk factors remained associated with HCV seroprevalence except lack of formal education and HBsAg positivity. The only new association was a history of alcohol use (OR = 5.56, 95% CI 2.74–11.30). The %OR change from the main meta‐analyses was > 50% for ages 21–64 (OR = 3.01, 95% CI 1.91–4.74) and 65+ (OR = 21.35, 95% CI 11.54–39.51). Details are in Table S5.

In sensitivity analyses using studies conducted across all age groups (*n* = 29): most analysed risk factors remained significantly associated with HCV seroprevalence, while history of ever medical injection (OR = 1.48, 95% CI 1.15–1.89) emerged as a risk factor. Formal education, HIV and HBsAg status and history of medical operation became insignificant. There was no %OR change for age 65+, but the change was 31% for age group 21–64 years (OR = 2.31, 95% CI 1.20–4.44). Details are available in Table S6.

In the third sensitivity analysis using 29 studies conducted since 2015, the risk factors that remained associated with HCV seroprevalence were age 65+, being married and divorced, PLWH, blood transfused, family history of HCV and scarification; while being aged 21–64, not formally educated, HBsAg status and medical operation were not associated. A higher %OR change (52%) was detected for those with no formal education (Table S7).

## 4 Discussion

This is the first comprehensive systematic review and meta‐analysis of risk factors of HCV among the general population in SSA. We found that different healthcare‐associated factors, scarification practices, family history of HCV and having a history of IDU were associated with higher HCV seroprevalence among the general population in SSA. Additionally, populations such as older individuals, those not formally educated, being married or divorced, PLWH and carriers of HBsAg had higher odds of HCV seropositivity. The association was weak (OR = 1.2–1.4) for history of medical operation and scarification; moderate (OR = 1.5–2.9) for no formal education, being married, family history of HCV, HBsAg positive, PLWH, history of hospitalisation and blood transfusion and strong (OR ≥ 3.0) for age 65+, being divorced and history of IDU with large uncertainty.

In all analyses, the odds of HCV seropositivity were higher for age 65+. When we restricted the analysis to high‐quality studies, the association strengthened (%OR > 50%) for both age groups (21–64 and 65+). In analyses restricting to studies that only included samples of all‐age groups and reported HCV seroprevalence across the age groups, age group 21–64 and 65+ remained significantly associated and the %OR for the age group 21–64 increased by 31% from the main analyses. In this analysis, history of medical injection (OR = 1.48) was significantly associated with HCV seropositivity, which might be due to analyses by age group better capturing the contribution of lifetime exposure to unsafe medical injections, avoiding the dilution effect of including estimates from younger people born after the improvement of medical practices safety.

When the analysis was restricted to the most recent studies (since 2015), the association for the age group 21–64 became insignificant, while it remained unchanged for age 65+. These may be explained by the recent reduction of injection‐associated iatrogenic infections [20] due to the introduction of autodestructed/smart syringes or increased access to new syringes and needles in many SSA countries [21, 22].

The higher odds of HCV seroprevalence among PLWH and HBsAg‐positive individuals may imply shared routes of transmission, which underscores the importance of integrating HCV elimination programmes with the HIV and hepatitis B programmes. However, in sensitivity analyses, the association with HBsAg and HIV was lost, which might be related to the higher effect of hepatitis B and HIV sexual transmissions than HCV. On the other hand, the higher odds of HCV associated with poor educational background and different scarification practices could be due to a lack of knowledge related to risk factors for disease transmission and infectious control practices, which could be improved through expanded awareness creation using health education [4, 23].

History of alcohol use was not associated with HCV infection in the main analysis but significantly associated in the sensitivity analysis including only high‐quality studies. Alcohol use is not directly associated with HCV transmission, but it might increase risk behaviours such as IDU, incarcerations, homelessness, unprotected sexual contacts and frequent healthcare visits [24, 25, 26].

We detected variation in risk factors across SSA subregion that might be related to differences in healthcare safety practices and community cultures. In Eastern Africa, history of hospitalisation and blood transfusion were associated with increased odds of HCV seroprevalence, while blood transfusion was positively associated in Western Africa.

### 4.1 Comparison With Literature

A review by Sonderup et al. (2017) summarised that risk factors for HCV transmission in SSA are mainly through parenteral routes such as blood/blood product transfusion, needle stick injury to health workers and body piercing [3]. Another review published in 2002 reported increased HCV prevalence with increased age among the general population in SSA. The review also highlighted that the constant increase of HCV with age might be through ongoing and repeated exposure to unsafe medical care [27].

There is good evidence that historically the practice of blood product screening in SSA was problematic due to constraints in the cost of testing, lack of expertise and limited infrastructure [28]. Multiple multinational studies in SSA reported the continued use of poorly sensitive assays to screen blood before transfusion for HCV, limited access to HCV nucleic acid testing and evidence of HCV‐infected blood transfusions [29, 30]. There is evidence of blood supply safety improvements in SSA, but challenges remain [19, 31].

In SSA, historically, the reuse of unsterile medical injection materials was common and believed to have contributed to the increased burden of HCV in the region [32]. In 2004, WHO estimated that 17%–19% of medical injections in SSA were through reused syringes/needles [33]. A study conducted in 2000 reported unsafe medical injections attributed to 16.4% of incidence HCV cases in Africa [5]. Studies have evidenced that unsafe medical injections were associated with increased HCV burden in the region during mass vaccination programmes. For example, in Cameroon, the increased spread of HCV coincides with the mass vaccination and treatment campaign against trypanosomiasis and yaws between 1920 and 1960 [34, 35].

### 4.2 Strengths and Limitations

The search was comprehensive but did not include grey literature. The cross‐sectional nature of the included studies prevented us from demonstrating the temporal relationship between those risk factors and HCV exposure. Due to the nature of the available data and limited reporting of adjusted ORs within the studies included in the review, we were unable to control for confounding across each risk factor. There was a lack of consistency and differences in laboratory test methods and measurement of some risk factors across the included studies. Since we were only able to identify studies from 25 SSA countries representing approximately 75% of the general population in the region, these findings might not be generalisable to the whole of SSA. Particularly, there were few studies included in the Southern African region. Reported risk factors differed from country to country and given the small number of studies from each country we are unable to estimate country‐specific risk factors.

### 4.3 Implications for the SSA Regional HCV Elimination Programs

Our findings strengthen the evidence base demonstrating that healthcare‐associated factors and scarification practices are associated with increased HCV seroprevalence in SSA.

The lower risk we identified in the younger population and adults in the recent studies might be linked to the improvement in blood safety and a ban on the reuse of syringes for medical injections in many SSA countries [18, 19, 21]. However, evidence indicates the continued risk of bloodborne virus transmissions due to the transfusion of unscreened blood or blood screened using poor‐performance RDTs as the only screening assay in some SSA countries [29, 30]. Studies from Egypt reported that using safety‐engineered syringes reduces HCV infection, so improving quality‐adjusted life years, and markedly saving costs [36, 37]. Combined interventions such as banning the reuse of syringes and promotion of smart syringes use, using well‐qualified blood screening tests, universal sterilisation/disinfection of medical equipment, ongoing capacity building for healthcare workers and increased awareness raising of communities or service recipients can help to mitigate iatrogenic transmission of HCV [21, 23].

We identified that the history of IDU is associated with increased HCV seroprevalence among SSA general populations. The evidence is uncertain due to the limitation of community‐based studies to capture this subgroup of the population, but evidence from elsewhere is supportive of this [38]. HCV prevalence was estimated to be 21.8% among PWID in SSA, whereas 9% of the global population of PWID are estimated to be in SSA, or 0.3% of the general population in SSA [39]. Due to the low availability of needle exchange programs, harm reduction services and the criminalisation of IDU in most SSA countries [40, 41], this problem might fuel the HCV burden in SSA.

In considering certainty in the evidence, we reflected on relevant aspects of the GRADE framework [42]. In this review, certainty is limited due to concerns around potential biases—for example, risk factors were largely self‐reported, some of which (e.g., history of IDU) may be particularly susceptible to information bias. There was also notable heterogeneity (inconsistency) with high *I* ^2^ values for most risk factors assessed, together with observed variation in point estimates and confidence intervals across studies. Heterogeneity remained in sub‐group analyses by SSA region. There were some concerns regarding imprecision, with particularly wide confidence intervals for the analyses: 65+ years versus ≤ 20 years and history of IDU. There were no major concerns regarding indirectness, with most studies recruiting reasonably representative general population samples. For studies of prevalence, it is not clear if or how publication bias could manifest [43] and it was therefore not appropriate to assess it.

## 5 Conclusion

Our review reveals that the risk factors associated with increased HCV seroprevalence in SSA are multidimensional, ranging from healthcare‐associated factors to individual and cultural behaviours. In selected African countries, targeted screening of older adults and people exposed to a history of blood transfusion, medical injections or hospitalisation might be important for HCV case finding and subsequent linkage to treatment.

## Author Contributions

G.M.K., A.G.L., C.E.F., J.G.W. and M.H. conceptualised the study; G.M.K. screened titles, abstract, and full texts through blind dual screening by A.G.L., J.G.W., C.E.F., M.T.T. and T.S.A.; G.M.K. extracted the data with independent double extraction by M.T.T. and T.S.A.; G.M.K. analysed and interpreted the data with support from A.G.L., J.G.W., C.E.F. and M.H.; G.M.K. drafted the original manuscript supported by A.G.L., J.G.W., C.E.F. and MH; all authors edited and commented on the manuscript drafts and approved the final version of the manuscript.

## Conflicts of Interest

The authors declare no conflicts of interest.

## Supporting information


**Data S1:** jvh70065‐sup‐0002‐TableS1‐S8‐FigureS1‐S15.docx.

## Data Availability

All underlying data and their sources are available within the Data S1.

## Appendix A DESTINE NIHR Global Health Research Group

Professor John F. Dillon (University of Dundee), Professor Wondwossen Amogne (Addis Ababa University), Professor Peter Vickerman (University of Bristol), Professor Matthew Hickman (University of Bristol), Professor Ora Paltiel (Hadassah‐Hebrew University), Dr. Dawit Wolday (Ethiopian Public Health Institute/McMaster University), Dr. Aynishet Adane (University of Gondar), Mrs. Saro Abdella (University of Dundee/Ethiopian Public Health Institute), Dr. Zenahbezu Abay (University of Gondar), Dr. Workagegnehu Hailu (University of Gondar), Professor Tadesse Awoke (University of Gondar), Dr. Emebet Dagne (Jimma University), Dr. Elias Ali Yesuf (Jimma University), Dr. Josephine G. Walker (University of Bristol), Dr. Aaron G. Lim (University of Bristol), Dr. Clare E. French (University of Bristol), Dr. Andargachew Mulu (Armauer Hansen Research Institute), Melaku Tileku Tamiru (University of Dundee/Addis Ababa University), Atsbeha Gebreegziabxier Weldemariam (University of Dundee/Ethiopian Public Health Institute), Dr. Christie Cabral (University of Bristol), Dr. Obsie Baissa (Hadassah‐Hebrew University), Ms. Elizabeth Speakman (University of Dundee), Dr. Andrew Radley (NHS Tayside), Dr. Amy Malaguti (NHS Tayside), Dr. Sarah Inglis (University of Dundee), Ms. Meseret Yohannes (Addis Ababa University), Ms. Bruktait Taddele (Addis Ababa University), Dr. Hagos Abraha (Mekelle University), Dr. Mengistu Erkie (Addis Ababa University), Tesfa Sewunet Alamneh (University of Bristol/University of Gondar) and Getahun Molla Kassa (University of Bristol/University of Gondar).
