# Supplementary material for: Risk Factors for Hepatitis C Virus Among the General Population in Sub‐Saharan Africa—An Analysis of Systematic Review Data
Source: J Viral Hepat. 2025 Sep 1;32(10):e70065. doi: 10.1111/jvh.70065 (PMC12400790; doi:10.1111/jvh.70065)
Supplement: Supplementary file 1 — Data S1: jvh70065‐sup‐0002‐TableS1‐S8‐FigureS1‐S15.docx. [file JVH-32-0-s001.docx]

**Supplementary files**

**Risk-factors for hepatitis C virus among the general population in sub-Saharan Africa - an analysis of systematic review data**

Getahun Molla Kassa^1,2*^, Aaron G Lim^1^, Melaku Tileku Tamiru^3,4^, Tesfa Sewunet Alamneh^1,2^, Peter Vickerman^1^, Emebet Dagne^5^, Andargachew Mulu^6^, Obsie Baissa^7^, Ora Paltiel^7^, John F Dillon^3^, Elias Ali Yesuf^8^, Matthew Hickman^1,9^, Josephine G Walker^1^^, Clare E French^1,10^^, on behalf of the DESTINE NIHR *Global Health Research Group*

**Authors Affiliations**

^1^Population Health Sciences, Bristol Medical School, University of Bristol, Bristol, UK.

^2^Department of Epidemiology and Biostatistics, Institute of Public Health, College of Medicine and Health Sciences, University of Gondar, Gondar, Ethiopia.

^3^Division of Molecular and Clinical Medicine, School of Medicine, University of Dundee, Dundee, UK.

^4^Department of Pharmacology and Clinical Pharmacy, School of Pharmacy, College of Health Sciences, Addis Ababa University, Addis Ababa, Ethiopia.

^5^Department of Internal Medicine, Institute of Health, Jimma University, Jimma, Ethiopia.

^6^Armauer Hansen Research Institute, Addis Ababa, Ethiopia

^7^Department of Hematology and Braun School of Public Health, Hadassah-Hebrew University Faculty of Medicine, Jerusalem, Israel.

^8^Department of Health Policy and Management, Institute of Health, Jimma University, Jimma, Ethiopia.

^9^National Drug and Alcohol Research Centre, University of New South Wales, Sydney, Australia.

^10^NIHR Health Protection Research Unit in Behavioural Science and Evaluation at University of Bristol, Bristol, UK.

^Joint senior authors

*Corresponding Author

**Email**: [getahun.kassa@bristol.ac.uk](mailto:getahun.kassa@bristol.ac.uk)

*Getahun Molla Kassa, Population Health Sciences, Bristol Medical School, Oakfield House, Oakfield Grove, BS8 2BN, Bristol, UK.*

Table of Contents

[Search strategy 3](#_Toc188622273)

[Reported HCV seroprevalence associated risk factors 4](#_Toc188622274)

[Re-grouping of risk factors 5](#_Toc188622275)

[Geographical distribution of all included studies on HCV risk factors 5](#_Toc188622276)

[Quality assessment for included studies 7](#_Toc188622277)

[Forest plots for each associated risk factor with HCV seroprevalence from the main meta-analyses 9](#_Toc188622278)

[Subgroup analysis using SSA subregions 22](#_Toc188622279)

[Sensitivity analyses results 25](#_Toc188622280)

[Characteristics of included studies 29](#_Toc188622281)

[References 39](#_Toc188622282)

# Search strategy

**Table s1:** Summary of search strategy.

| **Search #** | **Concept** | **Searching strategy** |
| --- | --- | --- |
| **#1** | **Epidemiology** | exp Epidemiology/ OR exp Morbidity/ OR exp Prevalence/ OR exp Incidence/ OR Epidemiology.tw,kw. OR Prevalence*.tw,kw. OR incidence*.tw,kw. OR Attack Rate*.tw,kw. OR epidemic*.tw,kw. OR frequency.tw,kw. OR morbidity.tw,kw. OR outbreak*.tw,kw. OR endemic*.tw,kw. or rate.tw,kw. |
| **#2** | **Risk factors** | exp Risk Factors/ OR Risk Factor*.tw,kw. OR Factor, Risk.tw,kw. OR Health Correlate*.tw,kw. OR Associated Factor*.tw,kw. OR Predictor*.tw,kw. OR Determinant*.tw,kw. |
| **#3** | **Hepatitis C Virus** | exp Hepatitis C/ OR exp Hepacivirus/ OR exp Hepatitis C Antibodies/ OR exp Hepatitis C Antigens/ OR exp Hepatitis C, Chronic/ OR hepatitis c virus*.tw,kw. OR hepatitis c.tw,kw. OR HCV.tw,kw. OR hep C.tw,kw. OR hepacivirus*.tw,kw. OR Hepatitis C Virus Antibodies.tw,kw. OR HCV Antibodies.tw,kw. OR Anti?HCV Antibodies.tw,kw. OR Anti?Hepatitis C Virus Antibodies.tw,kw. OR Hepatitis C Antigen.tw,kw. OR Chronic Hepatitis C.tw,kw. |
| **#4** | **Africa** | exp Africa/ or exp Africa, Eastern/ or exp Africa, Western/ or exp Africa, Southern/ or exp "Africa South of the Sahara"/ or exp Africa, Northern/ or exp Africa, Central/ OR Africa.tw,kw. OR sub?Sahara Africa.tw,kw. OR Algeria.tw,kw. OR Egypt.tw,kw. OR Libya.tw,kw. OR Morocco.tw,kw. OR Sudan.tw,kw. OR Tunisia.tw,kw. OR Burundi.tw,kw. OR Comoros.tw,kw. OR Djibouti.tw,kw. OR Eritrea.tw,kw. OR Ethiopia.tw,kw. OR Kenya.tw,kw. OR Madagascar.tw,kw. OR Mozambique.tw,kw. OR Malawi.tw,kw. OR Mauritius.tw,kw. OR Rwanda.tw,kw. OR Seychelles.tw,kw. OR Somalia.tw,kw. OR South Sudan.tw,kw. OR Tanzania.tw,kw. OR Uganda.tw,kw. OR Zambia.tw,kw. OR Zimbabwe.tw,kw. OR Benin.tw,kw. OR Burkina Faso.tw,kw. OR Cape Verde.tw,kw. OR Cabo Verde.tw,kw. OR Gambia.tw,kw. OR Ghana.tw,kw. OR Guinea.tw,kw. OR Guinea Bissau.tw,kw. OR Ivory Coast.tw,kw. OR Cote divoire.tw.kw. OR Liberia.tw,kw. OR Mali.tw,kw. OR Mauritania.tw,kw. OR Niger.tw,kw. OR Nigeria.tw,kw. OR Senegal.tw,kw. OR Sierra Leone.tw,kw. OR Togo.tw,kw. OR Angola.tw,kw. OR Cameroon.tw,kw. OR Central African Republic.tw,kw. OR Chad.tw,kw. OR Congo.tw,kw. OR DR Congo.tw,kw. OR Democratic Republic of the Congo.tw,kw. OR Republic of the Congo.tw,kw. OR Equatorial Guinea.tw,kw. OR Gabon.tw,kw. OR (Sao Tome and Principe).tw,kw. OR (Sao Tome & Principe).tw.kw. OR Botswana.tw,kw. OR Eswatini.tw,kw. OR Lesotho.tw,kw. OR Namibia.tw,kw. OR South Africa.tw,kw. |
| **#5** | **Final search** | **(#1 OR #2) AND #3 AND #4 limit to humans only** |

**exp:** expanded search (MeSH term); **tw:** text word (written in the title or abstract); and **kw:** keywords (written in the keywords)

# Reported HCV seroprevalence associated risk factors

Complete list of all sociodemographic, economic, behavioural, and direct and indirect HCV transmissions factors reported from the included studies are available in **Table s2**. The most reported risk factors were sex, age, education status, marital status, hepatitis B virus surface antigen (HBsAg) status, history of blood or blood product transfusion, and body scarification or tattooing.

**Table s2:** Complete list of all reported risk factors among the general population in SSA.

| **Classification** | **Risk factors** |
| --- | --- |
| Sociodemographic | Age*, sex*, educational status*, marital status*, urban/rural residency*, religion, number of people in the families, ethnicity, living in conflict zone, region, and community size. |
| Economic | Occupation*, wealth index*, having health insurance, and housing status. |
| Comorbidities | HIV*, diabetic, high blood pressure, tuberculosis, renal failure, hepatitis B virus*, human papillomavirus (HPV), cancer, liver diseases, cardiac diseases, history of venereal diseases or sexual transmitted infections, history of schistosomiasis, human T-lymphotropic virus (HTLV), genital ulcer, and obesity (body mass index). |
| Healthcare related | Blood/blood product transfusion*, medical injection*, contraceptive injection, vaccine scar, injection for trypanosomiasis prophylaxis or treatment, history of hospitalization*, minor/major medical operation/surgery*, and HBV vaccination status. |
| Behavioural | Injecting drug use*, alcohol use*, cigarette smoking, and khat chewing. |
| Sexual | Age at first sex, history of multiple sexual partners*, ever brought/sold sex, condom use, and living in couple. |
| Traditional/harmful practices | Scarification* (Traditional operation, scarification, tattooing, facial/tribal marking, skin pricing procedures, ear pricing, eyebrow incision), uvulectomy, bloodletting, shared sharps/piercing materials or injury with sharp/piercing materials*, traditional male circumcision*, female genital mutilation, place of male circumcision or female genital mutilation, dental extraction*, and shared toothbrush. |
| HCV exposure history | Family history of hepatitis C*, family history of liver disease, contact history with known HCV or liver patients, and care for hepatitis patient. |
| Maternal health | Number of births, place of birth/delivery, pregnancy status, history of dilatation and curettage, and history of abortion. |

*Risk factors reported from five or more studies

# Re-grouping of risk factors

We regrouped related risk factors together (**Table s3)**.

**Table s3:** List of re-grouped risk factors or categories

| **Re-grouped name** | **Re-grouped variables or categories** |
| --- | --- |
| Divorced | Marital status of divorced, widowed, and separated |
| Married | Marital status of married and cohabitated |
| Never married | Marital status of single or never married |
| Blood transfusion | Any form of blood or blood product transfusions |
| Scarification | Any form of body scarification, tattooing, skin pricing, facial/tribal marking, ear pricing, and eyebrow incision |
| Probability sampling methods | Study subjects are recruited randomly such as using survey, simple random, systematic random, stratified random, random cluster, stratified-cluster, multi-stage random sampling methods or every study population has equal chance of participating in the study |
| Non-probability sampling methods | Study subject has no equal chance of selection/participation in the study such as snowball/referral, quota, convenience, purposive, volunteer, and judgemental sampling methods |

# Geographical distribution of all included studies on HCV risk factors

**Figure s1** showed the country level geographical distribution of all included studies from 25 SSA countries. The highest number are from Nigeria (n=22, 23.9%), Cameroon (n=15, 16.3%) and Ethiopia (n=11, 12.0%). The rest are from Tanzania (n=8, 8.7%), Rwanda (n=5, 5.4%), Burkina Faso (n=4, 4.4%), Gabon (n=3, 3.3%), Madagascar (n=3, 3.3%), Central Africa Republic (n=2, 2.2%), Guinea Bissau (n=2, 2.2%), Kenya (n=2, 2.2%), Sudan (n=2, 2.2%), Benin (n=1, 1.1%), Burundi (n=1, 1.1%), DR Congo (n=1, 1.1%), Equatorial Guinea (n=1, 1.1%), Ghana (n=1, 1.1%), Guinea (n=1, 1.1%), Mayotte (n=1, 1.1%), Seychelles (n=1, 1.1%), Sierra Leone (n=1, 1.1%), Somalia (n=1, 1.1%), South Africa (n=1, 1.1%), Zimbabwe (n=1, 1.1%), and Republic of Congo (n=1, 1.1%).


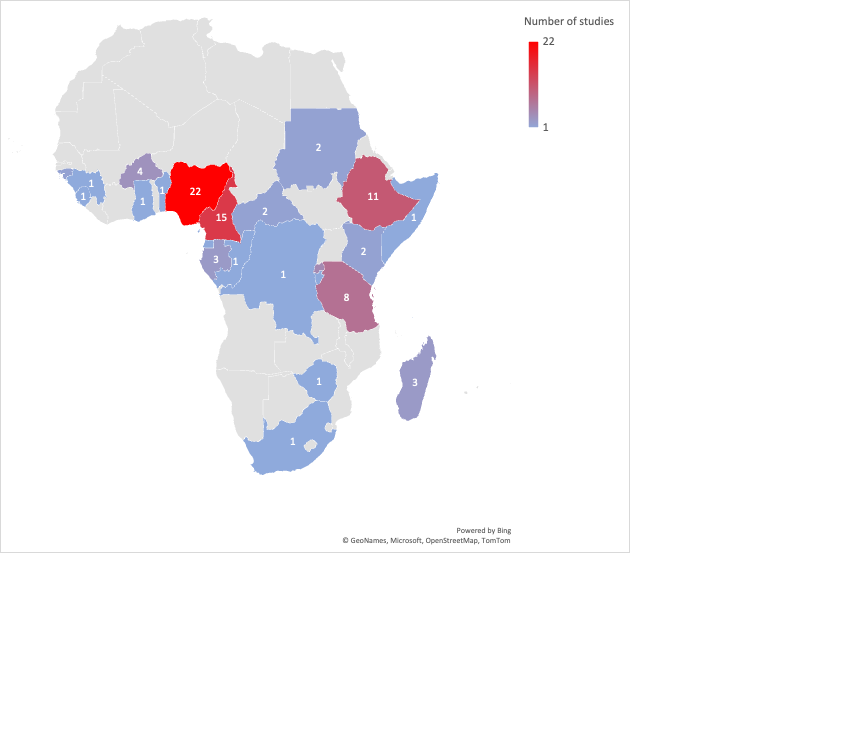


**Figure s1:** Geographical distribution of included studies (*Countries that have at least one study or dataset are colour shaded and the number of studies contributed is indicated in the center of each country*).

# Quality assessment for included studies

The Joanna Briggs Institute (JBI) quality assessment tool is the preferred tool to assess the quality of analytical cross-sectional studies (1) and we assess the methodological quality of the included studies using the JBI tool (2). This tool has nine quality assessment domains with a possible answer of either “yes”, “no”, “unclear”, or “not applicable”. We expect all studies should fulfil all the 9 domains and we didn’t use the answer “not applicable”. Finally, we sum-up all yeses and shows a score from 0-9 for each study. Studies scored eight or more yeses are grouped under high quality, six and seven yeses as moderate qualities, and below six yeses as poor quality. **Figure s2** depicts the overall quality assessment response respected to each JBI critical appraisal domains/questions. The overall JBI quality assessment score for the individual studies is available within **Table s8**.

Questions of Johanna Briggs Institute Critical Appraisal Tool for systematic reviews of prevalence studies.

D1. Was the sample frame appropriate to address the target population?

D2. Were study participants sampled in an appropriate way?

D3. Was the sample size adequate?

D4. Were the study subjects and the setting described in detail?

D5. Was the data analysis conducted with sufficient coverage of the identified sample?

D6. Were valid methods used for the identification of the condition?

D7. Was the condition measured in a standard, reliable way for all participants?

D8. Was there appropriate statistical analysis?

D9. Was the response rate adequate, and if not, was the low response rate managed appropriately?

**Figure s2**: Overall JBI quality assessment responses using the 9 critical appraise questions/domains to assesses prevalence studies.

# Forest plots for each associated risk factor with HCV seroprevalence from the main meta-analyses

**Forest plots captions**-- red vertical line (no effect line), green Vertical line (overall effect line), green diamond shape (commutative effect size (OR with 95% CI)), green horizontal line across the green diamond shape (95% prediction interval).

**Figure s3:** Forest plot for age 21-64 compared to age <20

**Figure s4:** Forest plot for age 65+ compared to age <20

**Figure s5:** Forest plot for not formal education as compared to secondary or above school attended

**Figure s6:** Forest plot for marital status of married compared to never married/single

** Figure s7:** Forest plot for marital status of divorced/widowed compared to never married/single

** Figure s8:** Forest plot for PLWH compared to people who were HIV negative

** Figure s9:** Forest plot for HBsAg positive compared to HBsAg negative

** Figure s10:** Forest plot for blood transfused compared to never transfused

** Figure s11:** Forest plot for individual with family history of HCV compared to those without

** Figure s12:** Forest plot for those with hospitalization history compared to never hospitalized

** Figure s13:** Forest plot for those with history of medical operation compared to never medically operated

** Figure s14:** Forest plot comparing those with scarification/tattooing with those not having scarification/tattooing

** Figure s15:** Forest plot for history of IDU compared to never IDU

# Subgroup analysis using SSA subregions

**Table s4**: Associated factors of HCV seroprevalence among the general population in the subregions of SSA.

| **Risk factors** | **Categories** | **Central-Africa** | | | **Eastern-Africa** | | | **Western-Africa** | | |
| --- | --- | --- | --- | --- | --- | --- | --- | --- | --- | --- |
|  |  | **HIV+/HCV-** | **OR (95% CI)** | **n, I2** | **HIV+/HCV-** | **OR (95% CI)** | **n, I2** | **HIV+/HCV-** | **OR (95% CI)** | **n, I2** |
| Gender/sex | Female | 977/17082 | Reference |  | 57676/924143 | Reference |  | 834/20521 | Reference |  |
|  | Male | 827/15473 | 1.06 (0.91-1.23) | 21, 40.48 | 28191/438202 | 0.97 (0.89-1.06) | 25, 80.12 | 567/18903 | 0.99 (0.84-1.17) | 26, 14.51 |
| Age in years | <20* | 29/6980 | Reference to 21-64 |  | 499/54913 | Reference to 21-64 |  | 149/7580 | Reference to 21-64 |  |
|  |  | 19/2186 | Reference to 64+ |  | 430/49622 | Reference to 64+ |  | __ | Reference to 64+ |  |
|  | 21-64 | 488/18067 | **5.33 (2.90-9.79)** | 11, 57.01 | 4530/179386 | 1.43 (0.79-2.59) | 17, 95.08 | 627/23360 | 0.96 (0.49-1.86) | 12, 76.90 |
|  | 65+ | 413/1730 | **25.23 (12.76-49.89)** | 7, 42.04 | 9564/49919 | **6.61 (1.81-24.12)** | 9, 98.67 | __ | Few studies |  |
| Education | Not formally educated | __ | Few studies | __ | 50/1173 | 1.26 (0.31-5.06), 7, 71.09 |  | __ | Few studies |  |
|  | Primary | __ | Few studies | __ | 52/1667 | 1.81 (0.54-2.58) | 7, 31.48 | __ | Few studies |  |
|  | Secondary or above | 60/996 | Reference |  | 19/1092 | Reference |  | 136/4039 | Reference |  |
| Marital status | Never married/single** | __ | Reference to married |  | 2939/184305 | Reference to married |  | 155/8708 | Reference to married |  |
|  |  | __ | Reference to divorced |  | 2933/183696 | Reference to divorced |  | __ | Reference to divorced |  |
|  | Married | __ | Few studies |  | 38663/765484 | **1.84 (1.23-2.75)** | **11, 97.11** | 533/15483 | **2.27 (1.32-3.91)** | **6, 67.79** |
|  | Divorced | __ | Few studies |  | 15786/198533 | **2.78 (1.36-5.70)** | **10, 99.05** | __ | Few studies |  |
| Residency | Urban | __ | Reference |  | 1554/78751 | Reference |  | __ | Reference |  |
|  | Rural | __ | Few studies |  | 2142/85642 | 1.48 (0.77-2.83) | 7, 93.73 | __ | Few studies |  |
| HIV | Negative | __ | Reference |  | 56485/1103347 | Reference |  | 108/6534 | Reference |  |
|  | Positive | __ | Few studies |  | 2552/29060 | **2.92 (1.76-4.84)** | **11, 97.64** | 16/470 | 4.22 (0.86-20.70) | 5, 83.39 |
| HBsAg | Negative | __ | Reference |  | 56468/1051863 | Reference |  | 869/29182 | Reference |  |
|  | Positive | __ | Few studies |  | 1274/33148 | 1.41 (0.72-2.77) | 12, 96.90 | 103/2416 | **1.99 (1.08-3.67)** | 14, 93.53 |
| History of multiple sexual partners | No | __ | Reference |  | 24650/443441 | Reference |  | 262/4461 | Reference |  |
|  | Yes | __ | No studies |  | 756/17278 | 1.02 (0.83-1.27) | 7, 54.83 | 147/1438 | 1.84 (0.82-4.13) | 7, 82.87 |
| Blood transfused | No | __ | Reference |  | 57849/1106248 | Reference |  | 266/10060 | Reference |  |
|  | Yes | __ | Few studies |  | 1591/26848 | **2.01 (1.18-3.43)** | **12, 96.84** | 52/907 | **2.05 (1.15-3.65)** | **9, 60.62** |
| History of dental extraction | No | __ | Reference |  | 30/1864 | Reference |  | __ | Reference |  |
|  | Yes | __ | No studies |  | 53/2006 | 1.91 (0.77-4.73) | 5, 52.16 | __ | No studies |  |
| Family history of HCV | No | __ | Reference |  | 56870/1249600 | Reference |  | __ | Reference |  |
|  | Yes | __ | No studies |  |  | **1.52 (1.14-2.03)** | **6, 92.45** | __ | Few studies |  |
| History of hospitalization | No | __ | Reference |  | 144/ 1402 | Reference |  | __ | Reference |  |
|  | Yes | __ | Few studies |  | **305/2091** | **2.06 (1.26-3.37)** | **5, 0.00** | __ | Few studies |  |
| History of medical operation/procedures | No | __ | Reference |  | 56042/1071195 | Reference |  | __ | Reference |  |
|  | Yes | __ | Few studies |  | 3398/59463 | 1.26 (0.98-1.63) | 10, 91.37 | __ | Few studies |  |
| Scarification/Tattooing | No | __ | Reference |  | 20899/400732 | Reference |  | 238/6551 | Reference |  |
|  | Yes | __ | Few studies |  | 4729/68926 | 1.84 (1.00-3.42) | 8, 98.28 | 80/1766 | 1.24 (0.95-1.62) | 8, 0.00 |

HCV (hepatitis C virus), HCV+ (hepatitis C virus antibody positive), HCV- (hepatitis C virus antibody negative), OR (odds ratio), CI (confidence interval), HIV (human immunodeficiency virus), HBsAg (hepatitis B virus surface antigen), n (number of studies), I2 (index of heterogeneity statistics), few studies (number of studies are <5), %OR (percent OR change).

*Some studies have data only for age <20 and 21-64 to compare and others for age 65+ to compare to age <20, because there were some studies conducted only among those aged <65 years and others conducted in all age groups. **some studies only have data for never married/single and married to compare whilst some others have also data for divorced.

# Sensitivity analyses results

**We ran three sensitivity analyses:**

1. Using high quality studies (**Table s5**),
2. Using studies reported HCV seroprevalence across all age groups (**Table s6**), and
3. Using studies conducted since 2015 (**Table s7**)

**Table s5**: Associated factors of HCV transmission among the general population in SSA from the meta-analysis of high-quality studies.

| **Risk factors** | **Categories** | **HCV+** | **HCV-** | **OR (95% CI)** | **n, I^2^** | **%OR** |
| --- | --- | --- | --- | --- | --- | --- |
| Gender/sex | Female | 58603 | 950527 | Reference |  |  |
|  | Male | 29100 | 463928 | 1.03 (0.96-1.11) | 39, 78.86 |  |
| Age in years | <20* | 616 | 66707 | Reference to age 21-64 |  |  |
|  |  | 414 | 52503 | Reference to age 65+ |  |  |
|  | 21-64 | 5543 | 216077 | **3.01 (1.91-4.74)** | **25, 93.85** | 70 |
|  | 65+ | 9989 | 51691 | **21.35 (11.54-39.51)** | 14, 93.50 | 82 |
| Education | Not formally educated | 593 | 13007 | 1.42 (0.85-2.39) | 10, 60.73 | 20 |
|  | Primary | 317 | 6600 | 0.97 (0.32-2.93) | 10, 92.75 |  |
|  | Secondary or above | 154 | 5786 | Reference |  |  |
| Marital status | Never married/single** | 3102 | 197870 | Reference to married |  |  |
|  |  | 3076 | 195467 | Reference to divorced |  |  |
|  | Married | 39236 | 787290 | **2.08 (1.57-2.77)** | 16, 94.99 | 9 |
|  | Divorced | 15815 | 199635 | **3.51 (2.01-6.12)** | 13, 98.50 | 10 |
| Residency | Urban | 1730 | 91626 | Reference |  |  |
|  | Rural | 2714 | 105060 | 1.27 (0.83-1.96) | 9, 93.45 |  |
| HIV/AIDS status | Negative | 56669 | 1119478 | Reference |  |  |
|  | Positive | 2545 | 29844 | **2.44 (1.32-4.54)** | 11, 98.80 | 12 |
| HBsAg status | Negative | 57265 | 1079096 | Reference |  |  |
|  | Positive | 1354 | 35445 | 1.31 (0.85-2.00) | 19, 93.46 | 21 |
| History of multiple sexual partners | No | 24877 | 446160 | Reference |  |  |
|  | Yes | 884 | 18091 | 1.28 (0.95-1.74) | 9, 84.14 |  |
| History of medical injections | No | 408 | 4732 | Reference |  |  |
|  | Yes | 258 | 3595 | 1.21 (0.68-2.15) | 5, 77.50 |  |
| Blood transfused | No | 58450 | 1117837 | Reference |  |  |
|  | Yes | 1671 | 27943 | **1.98 (1.3-3.01)** | **16, 96.10** | 9 |
| Family history of HCV | No | 56868 | 1252943 | Reference |  |  |
|  | Yes | 2207 | 33577 | **1.55 (1.18-2.02)** | 6, 91.68 | 2 |
| History of medical operation/procedures | No | 56053 | 1074536 | Reference |  |  |
|  | Yes | 3398 | 59761 | **1.32 (1.02-1.72)** | **9, 92.83** | 3 |
| Scarification/Tattooing | No | 21133 | 403026 | Reference |  |  |
|  | Yes | 4925 | 69079 | **1.44 (1.10-1.88)** | 11, 90.91 | 12 |
| Alcohol use | No | 37 | 4211 | Reference |  |  |
|  | Yes | 31 | 579 | **5.56 (2.74-11.30)** | 5, 27.59 | 147 |

HCV (hepatitis C virus), HCV+ (hepatitis C virus antibody positive), HCV- (hepatitis C virus antibody negative), OR (odds ratio), CI (confidence interval), HIV (human immunodeficiency virus), HBsAg (hepatitis B virus surface antigen), n (number of studies), I2 (index of heterogeneity statistics), %OR (percent OR change compared to the main analyses).

*Some studies have data only for age <20 and 21-64 to compare and others for age 65+ to compare to age <20, because there were some studies conducted only among those aged <65 years and others conducted in all age groups. **some studies only have data for never married/single and married to compare whilst some others have also data for divorced.

Because there were fewer than five studies, we did not assess the effect-size of history of hospitalization, IDU, male circumcision, dental extraction, and shared/injured by sharps materials. Of those 46 removed studies more than half were from Nigeria (15), Cameroon (8), and Tanzania (5).

**Table s6**: Associated factors of HCV seroprevalence among the general population in SSA from the meta-analysis using studies that reported HCV seroprevalence across all age groups.

| **Risk factors** | **Categories** | **HCV+** | **HCV-** | **OR (95% CI)** | **n, I2** | **%OR** |  |
| --- | --- | --- | --- | --- | --- | --- | --- |
| Gender/sex | Female | 58353 | 924376 | Reference |  |  |  |
|  | Male | 28732 | 441490 | 0.98 (0.91-1.06) | 27, 80.60 |  |  |
| Age in years | <20* | 458 | 52918 | Reference to age 21-64 |  |  | |
|  |  | 414 | 52503 | Reference to age 65+ |  |  | |
|  | 21-64 | 4735 | 183582 | **2.31 (1.20-4.44)** | 19, 95.64 | 31 | |
|  | 65+ | 9989 | 51691 | **11.75 (5.52-25.05)** | 19, 96.21 | 0 | |
| Education | Not formally educated | 110 | 1003 | 0.91 (0.29-2.91) | 5, 68.98 | 49 |  |
|  | Primary | 139 | 1374 | 0.63 (0.19-2.09) | 5, 76.63 |  |  |
|  | Secondary or above | 102 | 1822 | Reference |  |  |  |
| Marital status | Never married/single** | 2956 | 187766 | Reference to married |  |  |  |
|  |  | 2940 | 185649 | Reference to divorced |  |  |  |
|  | Married | 38790 | 768427 | **2.20 (1.50-3.22)** | 10, 97.60 | 15 |  |
|  | Divorced | 15923 | 198146 | **5.36 (2.73-10.52)** | 7, 99.14 | 67 |  |
| HIV/AIDS status | Negative | 56504 | 1100572 | Reference |  |  |  |
|  | Positive | 2525 | 28748 | 1.74 (0.88-3.41) | 7, 99.03 | 37 |  |
| HBsAg status | Negative | 56712 | 1057132 | Reference |  |  |  |
|  | Positive | 1309 | 33629 | 1.19 (0.74-1.91) | 13, 95.09 | 28 |  |
| History of multiple sexual partners | No | 24737 | 443406 | Reference |  |  |  |
|  | Yes | 748 | 16761 | 1.13 (0.78-1.65) | 5, 89.71 |  |  |
| History of medical injections | No | 463 | 4371 | Reference |  |  |  |
|  | Yes | 627 | 3689 | **1.48 (1.15-1.89**) | 6, 40.98 | 16 |  |
| Blood transfused | No | 58752 | 1111577 | Reference |  |  |  |
|  | Yes | 1740 | 27592 | **1.44 (1.11-1.87)** | 12, 90.57 | 20 |  |
| Family history of HCV | No | 56864 | 1249194 | Reference |  |  |  |
|  | Yes | 2199 | 33211 | **1.43 (1.12-1.83)** | 5, 91.08 | 6 |  |
| History of medical operation/procedures | No | 56037 | 1070785 | Reference |  |  |  |
|  | Yes | 3398 | 59417 | 1.27 (0.98-1.64) | 8, 93.36 | 1 |  |
| Scarification | No | 21179 | 399248 | Reference |  |  |  |
|  | Yes | 5077 | 68167 | **1.34 (1.06-1.70)** | 9, 90.52 | 4 |  |

HCV (hepatitis C virus), HCV+ (hepatitis C virus antibody positive), HCV- (hepatitis C virus antibody negative), OR (odds ratio), CI (confidence interval), HIV (human immunodeficiency virus), HBsAg (hepatitis B virus surface antigen), n (number of studies), I2 (index of heterogeneity statistics), %OR (percent OR change compared to the main analyses).

**some studies only have data for never married/single and married to compare whilst some others have also data for divorced.

We did not assess the association of HCV seroprevalence with history of hospitalization, urban/rural residency, male circumcision, IDU, dental extraction, Shared/injured by sharps materials, and alcohol use due to there being fewer than five studies.

**Table s7:** Associated factors of HCV transmission among the general population in SSA from the meta-analysis using studies conducted since 2015.

| **Risk factors** | **Categories** | **HCV+** | **HCV-** | **OR (95% CI)** | **n, I2** | **%OR** |
| --- | --- | --- | --- | --- | --- | --- |
| Gender/sex | Female | 57414 | 918115 | Reference |  |  |
|  | Male | 27975 | 434508 | 0.98 (0.89-1.07) | 23, 83.32 |  |
| Age in years | <20* | 361 | 49294 | Reference for age 21-64 |  |  |
|  |  | 321 | 47139 | Reference for age 65+ |  |  |
|  | 21-64 | 4144 | 173189 | 1.15 (0.49-2.73) | 10, 95.91 | 35 |
|  | 65+ | 9433 | 49386 | **11.86 (3.07-45.80)** | 6, 98.14 | 1 |
| Education | Not formally educated | 50 | 1548 | 0.85 (0.23-3.09) | 8, 59.17 | 52 |
|  | Primary | 33 | 1967 | 0.62 (0.20-1.90) | 8, 61.01 |  |
|  | Secondary or above | 48 | 2207 | Reference |  |  |
| Marital status | Never married/single** | 2935 | 186160 | Reference for married |  |  |
|  |  | 2925 | 184652 | Reference for divorced |  |  |
|  | Married | 38655 | 765511 | **2.26 (1.47-3.46)** | **11, 97.48** | 18 |
|  | Divorced | 15781 | 198273 | **4.53 (2.26-9.12)** | **9, 98.92** | 41 |
| HIV/AIDS status | Negative | 56471 | 1101439 | Reference |  |  |
|  | Positive | 2530 | 28566 | **3.13 (1.74-5.64)** | **10, 98.29** | 13 |
| HBsAg status | Negative | 56732 | 1059720 | Reference |  |  |
|  | Positive | 1299 | 33585 | 1.07 (0.72-1.59) | 16, 90.01 | 35 |
| History of multiple sexual partners | No | 24771 | 446403 | Reference |  |  |
|  | Yes | 17648 | 18406 | 1.08 (0.82-1.44) | 10, 69.81 |  |
| Blood transfused | No | 57920 | 1109362 | Reference |  |  |
|  | Yes | 1614 | 27225 | **1.90 (1.21-2.98)** | **12, 95.65** | 5 |
| Family history of HCV | No | 56903 | 1253267 | Reference |  |  |
|  | Yes | 2207 | 33592 | **1.52 (1.17-1.96)** | **7, 89.27** | 0 |
| History of medical operation/procedures | No | 56050 | 1072645 | Reference |  |  |
|  | Yes | 3395 | 59485 | 1.22 (0.96-1.54) | 9, 90.80 |  |
| Scarification | No | 20824 | 398221 | Reference |  |  |
|  | Yes | 4676 | 66727 | **1.56 (1.17-2.09)** | **8, 89.26** | 21 |

HCV (hepatitis C virus), HCV+ (hepatitis C virus antibody positive), HCV- (hepatitis C virus antibody negative), OR (odds ratio), CI (confidence interval), HIV (human immunodeficiency virus), HBsAg (hepatitis B virus surface antigen), n (number of studies), I2 (index of heterogeneity statistics), %OR (percent OR change compared to the main analyses).

*Some studies have data only for age <20 and 21-64 to compare and others for age 65+ to compare to age <20, because there were some studies conducted only among those aged <65 years and others conducted in all age groups. **some studies only have data for never married/single and married to compare whilst some others have also data for divorced.

We did not assess the effect size of residency, male circumcisions, medical injection, IDU, dental extraction, hospitalization, shared/injured by sharps materials, and alcohol use since there were fewer than five studies.

# Characteristics of included studies

**Table s8:** Characteristics of included studies (3-94)

| **Study ID** | **Title of the article** | **Country** | **Study Design** | **Study population** | **Sampling methods** | **HCV tested by** | **JBI-Quality** |
| --- | --- | --- | --- | --- | --- | --- | --- |
|  |  | **Central-Africa** |  |  |  |  |  |
| Tazinkeng 2022 | Factors associated with hepatitis B and C among adults in Buea, Cameroon: A community-based cross-sectional study | Cameroon | Cross-sectional | Mixed group | Sequential | RDT | High |
| Njouom 2018 | The burden of hepatitis C virus in Cameroon: Spatial epidemiology and historical perspective | Cameroon | Cross-sectional | Adult | Stratified Cluster | ELISA | High |
| Nansseu 2017 | Sero-epidemiology of human immunodeficiency virus, hepatitis B virus and hepatitis C virus: a cross-sectional survey in a rural setting of the West region of Cameroon | Cameroon | Cross-sectional | Mixed group | Voluntary | RDT | High |
| Nerrienet 2005 | Hepatitis C virus infection in cameroon: A cohort-effect | Cameroon | Cross-sectional | Mixed group | Unspecified | ELISA | High |
| Njouom 2003 | High Rate of Hepatitis C Virus Infection and Predominance of Genotype 4 Among Elderly Inhabitants of a Remote Village of the Rain Forest of South Cameroon | Cameroon | Cross-sectional | Mixed group | Samples from a representative group | ELISA | High |
| Delaporte 1994 | Hepatitis C in remote populations of southern Cameroon | Cameroon | Cross-sectional | Mixed group | Unspecified | ELISA | High |
| Ngatchu 1992 | Seroprevalence of anti-HCV in an urban child population: a pilot survey in a developing area, Cameroon | Cameroon | Cross-sectional | Children | Systematic Random | ELISA | High |
| Basimane-Bisimwa 2023 | Seroprevalence and molecular characterization of viral hepatitis and HIV co-infection in the Central African Republic | Central Africa Republic | Cross-sectional | Mixed group | Survey | ELISA | High |
| Fretz 1995 | HCV infection in a rural population of the Central African Republic (CAR): evidence for three additional subtypes of genotype 4 | Central Africa Republic | Cross-sectional | Mixed group | All area included | ELISA | High |
| Basaras 1999 | Seroprevalence of hepatitis B and C, and human immunodeficiency type 1 viruses in a rural population from the Republic of Equatorial Guinea | Equatorial Guinea | Cross-sectional | Mixed group | Unspecified | ELISA | High |
| Njouom 2012 | Phylogeography, risk factors and genetic history of hepatitis C virus in Gabon, central Africa | Gabon | Cross-sectional | Mixed group | Random | ELISA | High |
| Ndong-Atome 2008 | High prevalence of hepatitis C virus infection and predominance of genotype 4 in rural Gabon | Gabon | Cross-sectional | Mixed group | Survey | ELISA | High |
| Delaporte 1993 | High level of hepatitis C endemicity in Gabon, equatorial Africa | Gabon | Cross-sectional | Mixed group | Voluntary | ELISA | High |
| Kamdem 2019 | Negative Association of Interleukin-33 Plasma Levels and Schistosomiasis Infection in a Site of Polyparasitism in Rural Cameroon | Cameroon | Cross-sectional | Children | Voluntary | RDT | Moderate |
| Agbor 2018 | Prevalence of anti-hepatitis C antibodies and its co-infection with HIV in rural Cameroon | Cameroon | Cross-sectional | Mixed group | Consecutive | ELISA | Moderate |
| Biwole-Sida 2015 | Carriage prevalence of HCV markers in the working environment (Cameroun) | Cameroon | Cross-sectional | Adult | Voluntary | RDT | Moderate |
| Pepin 2010 | Risk Factors for Hepatitis C Virus Transmission in Colonial Cameroon | Cameroon | Cross-sectional | Old | Convenience | ELISA | Moderate |
| Kowo 1995 | Prevalence of hepatitis C virus and other blood-borne viruses in Pygmies and neighbouring Bantus in southern Cameroon | Cameroon | Cross-sectional | Mixed group | Voluntary | ELISA | Moderate |
| Louis 1994 | High prevalence of anti-hepatitis C virus antibodies in a Cameroon rural forest area | Cameroon | Cross-sectional | Mixed group | Survey | ELISA | Moderate |
| Hogan 2016 | Epidemic History and Iatrogenic Transmission of Blood-borne Viruses in Mid-20th Century Kinshasa | DR Congo | Cross-sectional | Old | Convenience | ELISA | Moderate |
| Cantaloube 2010 | Analysis of Hepatitis C Virus Strains Circulating inRepublic of the Congo | Republic of Congo | Cross-sectional | Mixed group | Random | ELISA | Moderate |
| Mbopi-Keou 2015 | [Prevalence and factors associated with HIV and viral hepatitis B and C in the city of Bafoussam in Cameroon] | Cameroon | Cross-sectional | Mixed group | Voluntary | Unspecified | Low |
| Mencarini 1991 | Prevalence of anti-HCV antibodies in Cameroon | Cameroon | Cross-sectional | Adult | Unspecified | ELISA | Low |
|  |  | **Eastern-Africa** |  |  |  |  |  |
| Ntagirabiri 2014 | Prevalence of hepatitis C virus in Burundi: A nationwide survey | Burundi | Cross-sectional | Mixed group | Cluster sampling | ELISA | High |
| Beykaso 2021 | Burden and Transmission Risks of Viral Hepatitis in Southern Ethiopia: Evidence Needed for Prevention and Control Measures | Ethiopia | Cross-sectional | Mixed group | Systematic random | ELISA | High |
| Biazin 2019 | DETERMINING SEROPREVALENCE OF HEPATITIS B AND C VIRUS INFEC-TIONS AND ASSOCIATED RISK FACTORS AMONG APPARENTLY HEALTHY MOTHERS IN ADDIS ABABA, ETHIOPIA | Ethiopia | Cross-sectional | Adult Women | Systematic random | ELISA | High |
| Woldegiorgis 2019 | Community-based sero-prevalence of hepatitis B and C infections in South Omo Zone, Southern Ethiopia | Ethiopia | Cross-sectional | Mixed group | Randomly | RDT | High |
| Abebe 2019 | SEROPREVALENCE OF HEPATITIS B VIRUS SURFACE ANTIGEN, ANTI-HEPATITIS C VIRUS ANTIBODY AND THEIR ASSOCIATED FACTORS AMONG MOTHERS LIVING IN HARAR, EASTERN ETHIOPIA | Ethiopia | Cross-sectional | Adult Women | Systematic random | ELISA | High |
| Abera 2017 | Community seroprevalence of hepatitis B, C and human immunodeficiency virus in adult population in gojjam zones, northwest Ethiopia | Ethiopia | Cross-sectional | Adult | Systematic random | RDT | High |
| Berhe 2007 | Intensity of Schistosoma Mansoni, Hepatitis B, Age, and Sex Predict Levels of Hepatic Periportal Thickening/Fibrosis (PPT/F): A Large-Scale Community-Based Study in Ethiopia | Ethiopia | Cross-sectional | Mixed group | Simple random | ELISA | High |
| Ayele 2002 | Higher Prevalence of Anti-HCV Antibodies AmongHIV-Positive Compared to HIV-Negative Inhabitants of Addis Ababa, Ethiopia | Ethiopia | Cross-sectional | Mixed group | Two-stage cluster | ELISA | High |
| Ly 2018 | The Prevalence of Hepatitis C Virus Antibody in HIV-Negative Persons in Kenya, 2007 | Kenya | Cross-sectional | Adult | Stratified cluster | ELISA | High |
| Kerubo 2015 | Hepatitis B, Hepatitis C and HIV-1 Coinfection in Two Informal Urban Settlements in Nairobi, Kenya | Kenya | Cross-sectional | Adult | None Probability | RDT | High |
| Ramarokoto 2008 | Seroprevalence of hepatitis C and associated risk factors in urban areas of Antananarivo, Madagascar | Madagascar | Cross-sectional | Mixed group | Cluster sampling | ELISA | High |
| Zeller 1997 | Prevalence of hepatitis C virus infection in general population in Madagascar | Madagascar | Cross-sectional | Mixed group | Simple random | ELISA | High |
| Brouard 2022 | Hepatitis B, C, and delta in the general population in Mayotte: hepatitis B as a major public health concern | Mayotte | Cross-sectional | Mixed group | Multi-stage | ELISA | High |
| Nisingizwe 2023 | The Cascade of Care for Hepatitis C Treatment in Rwanda: A Retrospective Cohort Study of the 2017–2019 Mass Screening and Treatment Campaign | Rwanda | Secondary data analysis of household survey | Mixed group | Survey | ELISA | High |
| Makuza 2020 | Role of unsafe medical practices and sexual behaviours in the hepatitis B and C syndemic and HIV co-infection in Rwanda: a cross-sectional study | Rwanda | Cross-sectional | Mixed group | Voluntary | RDT | High |
| Makuza 2019 | Risk factors for viral hepatitis C infection in Rwanda: results from a nationwide screening program | Rwanda | Cross-sectional | Mixed group | Voluntary | ELISA | High |
| Umutesi 2019 | Screening a nation for hepatitis C virus elimination: a cross-sectional study on prevalence of hepatitis C and associated risk factors in the Rwandan general population | Rwanda | Cross-sectional | Mixed group | Voluntary | ELISA | High |
| Bovet 1999 | Decrease in the prevalence of hepatitis B and a low prevalence of hepatitis C virus infections in the general population of the Seychelles | Seychelles | Cross-sectional | Adult | Stratified random | ELISA | High |
| Omer 2001 | The role of hepatitis B and hepatitis C viral infections in the incidence of hepatocellular carcinoma in Sudan | Sudan | Case-Control | Mixed group | Multi-stage | ELISA | High |
| Mazigo 2017 | Co-infection of Schistosoma mansoni/hepatitis C virus and their associated factors among adult individuals living in fishing villages, north-western Tanzania | Tanzania | Cross-sectional | Adult | Two-step random | RDT | High |
| Tess 2000 | SEROPREVALENCE OF HEPATITIS C VIRUS IN THE GENERAL POPULATION OF NORTHWEST TANZANIA | Tanzania | Cross-sectional | Adult | Random | ELISA | High |
| Kumalo 2022 | Undiagnosed Seroprevalence of Hepatitis B and C Virus Infections in the Community of Wolaita Zone, Southern Ethiopia | Ethiopia | Cross-sectional | Mixed group | Multi-stage | RDT | Moderate |
| Taye 2019 | Prevalence and associated risk factors of Hepatitis B and C virus infections among mothers in Jimma, South West Ethiopia: a community-based study | Ethiopia | Cross-sectional | Adult Women | Simple random | ELISA | Moderate |
| Morvan 1994 | Anti-HCV antibody prevalence among an asymptomatic population living in two villages in Madagascar | Madagascar | Cross-sectional | Mixed group | Random | ELISA | Moderate |
| Mudawi 2007 | Epidemiology of HCV Infection in GeziraState of Central Sudan | Sudan | Cross-sectional | Mixed group | Random | ELISA | Moderate |
| Froeschl 2021 | Hepatitis B, C and D virus prevalence in children and adults in Mbeya Region, Tanzania: results from a cohort study 2002 - 2009 | Tanzania | Cross-sectional | Mixed group | Random | ELISA | Moderate |
| Muro 2013 | Seroprevalence of hepatitis B and C viruses among children in Kilimanjaro Region, Tanzania | Tanzania | Cross-sectional | Children | Probability | ELISA | Moderate |
| Puato 2007 | Does HCV infection have a more favourable outcome in Tanzanian people?. Data from the Lugalawa study | Tanzania | Cross-sectional | Mixed group | Unspecified | ELISA | Moderate |
| Stark 2000 | Seroepidemiology of TT virus, GBC-C/HGV, and hepatitis viruses B, C, and E among women in a rural area of Tanzania | Tanzania | Cross-sectional | Adult Women | Survey | ELISA | Moderate |
| Miller 1998 | Seroprevalence of viral hepatitis in Tanzanian adults | Tanzania | Cross-sectional | Adult | Voluntary | ELISA | Moderate |
| Sentjens 2000 | Risk factors for HCV, HTLV-I/II and HIV infection in various population subsets in Ethiopia | Ethiopia | Cross-sectional | Adult | Unspecified | ELISA | Low |
| Frommel 1993 | A survey of antibodies to hepatitis C virus in Ethiopia | Ethiopia | Cross-sectional | Mixed group | Unspecified | ELISA | Low |
| Iradukunda 2020 | Risk factors associated with hepatitis B and C in rural population of Burera district, Rwanda | Rwanda | Cross-sectional | Mixed group | Voluntary | ELISA | Low |
| Bile 1992 | The risk for hepatitis A, B, and C at two institutions for children in Somalia with different socioeconomic conditions | Somalia | Cross-sectional | Children | Unspecified | Unspecified | Low |
| Laukmamm-Josten 1993 | HIV, hepatitis B and C seroprevalence in truckstops and near by communities in Tanzania | Tanzania | Cross-sectional | Mixed group | Voluntary | ELISA | Low |
|  |  | **Southern-Africa** |  |  |  |  |  |
| AbdoolKarim 1993 | Hepatitis C virus infection in urban and rural Natal/KwaZulu | South Africa | Cross-sectional | Mixed group | Random | ELISA | Moderate |
| Kallestrup 2003 | Low prevalence of hepatitis C virus antibodies in HIV-endemic area of Zimbabwe support sexual transmission as the major route of HIV transmission in Africa | Zimbabwe | Cross-sectional | Mixed group | Complete data | ELISA | Low |
|  |  | **Western-Africa** |  |  |  |  |  |
| Kpossou 2021 | [Seroprevalence of hepatitis C virus (HCV) antibodies and associated factors based on voluntary screening data collection in the general population in Benin in 2016] | Benin | Cross-sectional | Mixed group | Voluntary | RDT | High |
| Lingani 2020 | The changing epidemiology of hepatitis B and C infections in Nanoro, rural Burkina Faso: a random sampling survey | Burkina Faso | Cross-sectional | Children | Random | ELISA | High |
| Meda 2018 | Hepatitis B and C virus seroprevalence, Burkina Faso: a cross-sectional study | Burkina Faso | Cross-sectional | Adult | Stratified cluster | ELISA | High |
| Honge 2020 | Hepatitis B and C in the adult population of Bissau, Guinea-Bissau: a cross-sectional survey | Guinea Bissau | Cross-sectional | Mixed group | Random | ELISA | High |
| Plamondon 2007 | Hepatitis C Virus Infection in Guinea-Bissau: A SexuallyTransmitted Genotype 2 with Parenteral Amplification? | Guinea Bissau | Cross-sectional | Old | Voluntary | ELISA | High |
| Aliyu 2021 | The Burden of HIV, Hepatitis B and Hepatitis C by Armed Conflict Setting: The Nigeria AIDS Indicator and Impact Survey, 2018 | Nigeria | Cross-sectional | Mixed group | Two-stage cluster sampling | RDT | High |
| Malu 2020 | Prevalence of Hepatitis B Surface Antigen and Antibodies to Hepatitis C in the General Population of Benue State, Central Nigeria | Nigeria | Cross-sectional | Mixed group | Multi-staged | RDT | High |
| Okonkwo 2017 | Prevalence of hepatitis B, hepatitis C and human immunodeficiency viruses, and evaluation of risk factors for transmission: Report of a population screening in Nigeria | Nigeria | Cross-sectional | Mixed group | Multi-staged | ELISA | High |
| Eke 2016 | Seroprevalence and correlates of hepatitis c virus infection in secondary school children in Enugu, Nigeria | Nigeria | Cross-sectional | Univestiy/Secondary students | Multi-staged | ELISA | High |
| Onyekwere 2015 | Hepatitis B and C virus prevalence and association with demographics: report of population screening in Nigeria | Nigeria | Cross-sectional | Mixed group | Consecutive | ELISA | High |
| Quesada 2015 | Hepatitis C virus seroprevalence in the general female population from 8 countries | Nigeria | Cross-sectional | Adult Women | Random | ELISA | High |
| Forbi 2009 | Serological markers and risk factors for hepatitis B and hepatitis C viruses among students in a Nigerian university | Nigeria | Cross-sectional | Univestiy/Secondary students | Random | ELISA | High |
| Jeannel 1998 | Evidence for high genetic diversity and long-term endemicity of hepatitis C virus genotypes 1 and 2 in West Africa | Burkina Faso | Cross-sectional | Mixed group | Random | ELISA | Moderate |
| Martinson 1996 | Seroepidemiological Survey of Hepatitis B and C Virus Infections in Ghanaian Children | Ghana | Cross-sectional | Children | Stratified cluster | ELISA | Moderate |
| Japhet 2019 | HIV, HBV AND HCV PREVALENCE, CO-INFECTIONS, RISK FACTORS AND AWARENESS AMONG STUDENTS IN A NIGERIAN UNIVERSITY | Nigeria | Cross-sectional | Univestiy/Secondary students | Unspecified | ELISA | Moderate |
| Muhibi 2019 | Surveillance of anti-HCV antibody amongst in-school youth in a Nigeria university | Nigeria | Cross-sectional | Univestiy/Secondary students | Voluntary | ELISA | Moderate |
| Olalekan 2015 | Prevalence and risk factors for hepatitis B and C among sexually active undergraduates in southwestern Nigeria | Nigeria | Cross-sectional | Univestiy/Secondary students | Multi-stage | Unspecified | Moderate |
| Jemilohun 2014 | Prevalence of Hepatitis C virus antibody among undergraduates in Ogbomosho, South-Western Nigeria | Nigeria | Cross-sectional | Univestiy/Secondary students | Survey | RDT | Moderate |
| Pennap 2010 | Prevalence of hepatitis B and C virus infection among people of a local community in Keffi, Nigeria | Nigeria | Cross-sectional | Mixed group | Voluntary | ELISA | Moderate |
| Jeremiah 2009 | Seroepidemiology of Transfusion Transmissible Viral Infection among University Fresh Students in Port Harcourt, Nigeria | Nigeria | Cross-sectional | University/Secondary students | Survey | RDT | Moderate |
| Ouedraogo 2018 | Prevalence of HIV, hepatitis B and hepatitis C infection among potential participants to experimental vaccines trials in a rural area of Burkina Faso | Burkina Faso | Cross-sectional | Adult | Voluntary | RDT | Low |
| Diallo 1995 | Prevalence of exposure to aflatoxin and hepatitis B and C viruses in Guinea, West Africa | Guinea | Cross-sectional | Adult Male | Unspecified | ELISA | Low |
| Alkali 2023 | SEROEPIDEMIOLOGY OF HBV ANTIGEN AND ANTI-HCV AMONG GENERAL POPULATION IN A RURAL LOCAL IN BAUCHI STATE, NIGERIA | Nigeria | Cross-sectional | Mixed group | Survey | RDT | Low |
| Ugah 2021 | Epidemiology of hepatitis b virus, hepatitis C virus and human immunodeficiency virus co-infection among as symptomatic persons resident in Alex Ekwueme federal university Ndufu-alike | Nigeria | Cross-sectional | Mixed group | None Probability | ELISA | Low |
| Omolade 2018 | Prevalence of hepatitis C virus antibody among university students in Nigeria | Nigeria | Cross-sectional | University/Secondary students | Voluntary | RDT | Low |
| Ogefere 2016 | Potential risk factors and seroprevalence of hepatitis c virus infection among students in a tertiary institution in Southern Nigeria | Nigeria | Cross-sectional | University/Secondary students | Voluntary | ELISA | Low |
| Ramyil 2014 | Prevalence and coinfection of human immunodeficiency virus, hepatitis B virus and hepatitis c virus in mabudi rural community of Plateau, State, Nigeria | Nigeria | Cross-sectional | Mixed group | Voluntary | RDT | Low |
| Forbi 2010 | Urban-rural estimation of hepatitis C virus infection seroprevalence in north Central Nigeria | Nigeria | Cross-sectional | Mixed group | Unspecified | ELISA | Low |
| Mabayoje 2010 | PREVALENCE OF HEPATITIS B SURFACE ANTIGEN, HEPATITIS C AND HUMAN IMMUNODEFICIENCY VIRUS ANTIBODIES IN A POPULATION OF STUDENTS OF TERTIARY INSTITUTION IN NIGERIA | Nigeria | Cross-sectional | Univestiy/Secondary students | Voluntary | ELISA | Low |
| Nwankiti 2009 | Hepatitis C Virus infection in apparentenly healthy individuals with family history of diabetes in Vom, Plateau State Nigeria | Nigeria | Cross-sectional | Mixed group | Purposive | ELISA | Low |
| Ejele 2006 | Seroprevalence of hepatitis C virus in the Niger Delta of Nigeria | Nigeria | Cross-sectional | Mixed group | Consecutive | Unspecified | Low |
| Hodges 1998 | Seroprevalence of hepatitis markers; HAV, HBV, HCV and HEV amongst primary school children in Freetown, Sierra Leone | Sierra Leone | Cross-sectional | Children | Voluntary | Unspecified | Low |

Random (simple or systematic random sampling),

**DESTINE NIHR Global Health Research Group:** Professor John F Dillon (University of Dundee), Professor Wondwossen Amogne Degu (Addis Ababa University), Professor Peter Vickerman (University of Bristol), Professor Matthew Hickman (University of Bristol), Professor Ora Paltiel (Hadassah-Hebrew University), Dr Dawit Wolday (Ethiopian Public Health Institute/ McMaster University), Dr Aynishet Adane (University of Gondar), Mrs Saro Abdella (University of Dundee/Ethiopian Public Health Institute), Dr Zenahbezu Abay (University of Gondar), Dr Workagegnehu Hailu (University of Gondar), Professor Tadesse Awoke (University of Gondar), Dr Emebet Dagne (Jimma University), Dr Elias Ali Yesuf (Jimma University), Dr Josephine G Walker (University of Bristol), Dr Aaron G Lim (University of Bristol), Dr Clare E French (University of Bristol), Dr Andargachew Mulu (Armauer Hansen Research Institute), Melaku Tileku Tamiru (University of Dundee/Addis Ababa University), Atsbeha Gebreegziabxier Weldemariam (University of Dundee/Ethiopian Public Health Institute), Dr Christie Cabral (University of Bristol), Dr Obsie Baissa (Hadassah-Hebrew University), Ms Elizabeth Speakman (University of Dundee), Dr Andrew Radley (NHS Tayside), Dr Amy Malaguti (NHS Tayside), Dr Sarah Inglis (University of Dundee), Ms Meseret Yohannes (Addis Ababa University), Ms Bruktait Taddele (Addis Ababa University), Dr Hagos Abraha (Mekelle University), Dr Mengistu Erkie (Addis Ababa University), Tesfa Sewunet Alamneh (University of Bristol/University of Gondar), Getahun Molla Kassa (University of Bristol/University of Gondar).

**Roles of the funding source:** This research was funded by the NIHR (NIHR133208) using UK international development funding from the UK Government to support global health research. The views expressed in this publication are those of the author(s) and not necessarily those of the NIHR or the UK government.

# References

1. Ma LL, Wang YY, Yang ZH, Huang D, Weng H, Zeng XT. Methodological quality (risk of bias) assessment tools for primary and secondary medical studies: what are they and which is better? Mil Med Res. 2020;7(1):7.

2. Munn Z, Moola S, Lisy K, Riitano D, Tufanaru C. Methodological guidance for systematic reviews of observational epidemiological studies reporting prevalence and cumulative incidence data. Int J Evid Based Healthc. 2015;13(3):147-53.

3. Cantaloube J-F, Gallian P, Bokilo A, Jordier F, Biagini P, Attoui H, et al. Analysis of hepatitis C virus strains circulating in Republic of the Congo. JOURNAL OF MEDICAL VIROLOGY. 2010;82(4):562-7.

4. Morvan JM, Boisier P, Roux JF. Anti-HCV antibody prevalence among an asymptomatic population living in two villages in Madagascar. TRANSACTIONS OF THE ROYAL SOCIETY OF TROPICAL MEDICINE AND HYGIENE. 1994;88(6):657.

5. Beykaso G, Mulu A, Giday M, Berhe N, Selamu M, Mihret A, et al. Burden and Transmission Risks of Viral Hepatitis in Southern Ethiopia: Evidence Needed for Prevention and Control Measures. RISK MANAGEMENT AND HEALTHCARE POLICY. 2021;14:4843-52.

6. Njouom R, Siffert I, Texier G, Lachenal G, Tejiokem MC, Pepin J, et al. The burden of hepatitis C virus in Cameroon: Spatial epidemiology and historical perspective. JOURNAL OF VIRAL HEPATITIS. 2018;25(8):959-68.

7. Aliyu GG, Aliyu SH, Ehoche A, Dongarwar D, Yusuf RA, Aliyu MH, et al. The Burden of HIV, Hepatitis B and Hepatitis C by Armed Conflict Setting: The Nigeria AIDS Indicator and Impact Survey, 2018. ANNALS OF GLOBAL HEALTH. 2021;87(1):53.

8. Biwole-Sida M, Noah D, Eloumou AF, Dang I, Talla P, Malongue, et al. Carriage prevalence of HCV markers in the working environment (Cameroun). JOURNAL AFRICAIN D HEPATO-GASTROENTEROLOGIE. 2015;9(1):26-9.

9. Nisingizwe MP, Makuza JD, Janjua NZ, Bansback N, Hedt-Gauthier B, Serumondo J, et al. The Cascade of Care for Hepatitis C Treatment in Rwanda: A Retrospective Cohort Study of the 2017-2019 Mass Screening and Treatment Campaign. Viruses. 2023;15(3).

10. Lingani M, Akita T, Ouoba S, Nagashima S, Boua PR, Takahashi K, et al. The changing epidemiology of hepatitis B and C infections in Nanoro, rural Burkina Faso: a random sampling survey. BMC INFECTIOUS DISEASES. 2020;20(1):46.

11. Mazigo HD, Kepha S, Kaatano GM, Kinung'hi SM. Co-infection of Schistosoma mansoni/hepatitis C virus and their associated factors among adult individuals living in fishing villages, north-western Tanzania. BMC INFECTIOUS DISEASES. 2017;17(1):668.

12. Abera B, Adem Y, Yimer M, Mulu W, Zenebe Y, Mekonnen Z. Community seroprevalence of hepatitis B, C and human immunodeficiency virus in adult population in gojjam zones, northwest Ethiopia. VIROLOGY JOURNAL. 2017;14(1):21.

13. Woldegiorgis AE, Erku W, Medhin G, Berhe N, Legesse M. Community-based sero-prevalence of hepatitis B and C infections in South Omo Zone, Southern Ethiopia. PLOS ONE. 2019;14(12):e0226890.

14. Bovet P, Yersin C, Herminie P, Lavanchy D, Frei PC. Decrease in the prevalence of hepatitis B and a low prevalence of hepatitis C virus infections in the general population of the Seychelles. BULLETIN OF THE WORLD HEALTH ORGANIZATION. 1999;77(11):923-8.

15. Biazin H, Teshome S, Ayenew Z, Abebe T, Mihret A, Aseffa A, et al. Determining seroprevalence of Hepatitis B and C virus infections and associated risk factors among apparently healthy mothers in Addis Ababa, Ethiopia. Ethiop med j (Online). 2019;57(3):129-38.

16. Puato M, Migliorato I, Tirrito C, Ruvoletto M, Zanardo M, Pauletto P, et al. Does HCV infection have a more favourable outcome in Tanzanian people?. Data from the Lugalawa study. DIGESTIVE AND LIVER DISEASE. 2007;39(9):891-2.

17. Hogan CA, Iles J, Frost EH, Giroux G, Cassar O, Gessain A, et al. Epidemic History and Iatrogenic Transmission of Blood-borne Viruses in Mid-20th Century Kinshasa. The Journal of infectious diseases. 2016;214(3):353-60.

18. Mudawi HMY, Smith HM, Rahoud SA, Fletcher IA, Babikir AM, Saeed OK, et al. Epidemiology of HCV infection in Gezira state of central Sudan. JOURNAL OF MEDICAL VIROLOGY. 2007;79(4):383-5.

19. Ugah UI, Alo MN, Gloria UC. Epidemiology of hepatitis b virus, hepatitis C virus and human immunodeficiency virus co-infection among assymptomatic persons resident in Alex Ekwueme federal university Ndufu-alike. SCIENTIFIC AFRICAN. 2021;14.

20. Jeannel D, Fretz C, Traore Y, Kohdjo N, Bigot A, Pe Gamy E, et al. Evidence for high genetic diversity and long-term endemicity of hepatitis C virus genotypes 1 and 2 in West Africa. JOURNAL OF MEDICAL VIROLOGY. 1998;55(2):92-7.

21. Tazinkeng NN, Teuwafeu DG, Asombang AW, Agbor VN, Bloom SM, Nkhoma AN, et al. Factors associated with hepatitis B and C among adults in Buea, Cameroon: A community-based cross-sectional study. Liver international : official journal of the International Association for the Study of the Liver. 2022;42(11):2396-402.

22. Fretz C, Jeannel D, Stuyver L, Herve V, Lunel F, Boudifa A, et al. HCV infection in a rural population of the Central African Republic (CAR): evidence for three additional subtypes of genotype 4. JOURNAL OF MEDICAL VIROLOGY. 1995;47(4):435-7.

23. Honge BL, Olesen JS, Jensen MM, Jespersen S, da Silva ZJ, Rodrigues A, et al. Hepatitis B and C in the adult population of Bissau, Guinea-Bissau: a cross-sectional survey. Tropical medicine & international health : TM & IH. 2020;25(2):255-63.

24. Onyekwere CA, Hameed L. Hepatitis B and C virus prevalence and association with demographics: report of population screening in Nigeria. TROPICAL DOCTOR. 2015;45(4):231-5.

25. Meda N, Tuaillon E, Kania D, Tiendrebeogo A, Pisoni A, Zida S, et al. Hepatitis B and C virus seroprevalence, Burkina Faso: a cross-sectional study. BULLETIN OF THE WORLD HEALTH ORGANIZATION. 2018;96(11):750-9.

26. Froeschl G, Hoelscher M, Maganga LH, Kroidl I, Clowes P, Geis S, et al. Hepatitis B, C and D virus prevalence in children and adults in Mbeya Region, Tanzania: results from a cohort study 2002 - 2009. The Pan African medical journal. 2021;39:174.

27. Brouard C, Parenton F, Youssouf H, Chevaliez S, Gordien E, Jean M, et al. Hepatitis B, C, and delta in the general population in Mayotte: hepatitis B as a major public health concern. BMC INFECTIOUS DISEASES. 2022;22(1):716.

28. Kerubo G, Khamadi S, Okoth V, Madise N, Ezeh A, Ziraba A, et al. Hepatitis B, Hepatitis C and HIV-1 Coinfection in Two Informal Urban Settlements in Nairobi, Kenya. PLOS ONE. 2015;10(6):e0129247.

29. Delaporte E, Froment A, Dazza MC, Henzel D, Larouze B. Hepatitis C in remote populations of southern Cameroon. ANNALS OF TROPICAL MEDICINE AND PARASITOLOGY. 1994;88(1):97-8.

30. Nwankiti OO, Ndako JA, Echeonwu GO, Olabode AO, Nwosuh CI, Onovoh EM, et al. Hepatitis C Virus infection in apparentenly healthy individuals with family history of diabetes in Vom, Plateau State Nigeria. VIROLOGY JOURNAL. 2009;6:110.

31. Nerrienet E, Pouillot R, Lachenal G, Njouom R, Mfoupouendoun J, Bilong C, et al. Hepatitis C virus infection in cameroon: A cohort-effect. JOURNAL OF MEDICAL VIROLOGY. 2005;76(2):208-14.

32. Plamondon M, Labbe A-C, Frost E, Deslandes S, Alves AC, Bastien N, et al. Hepatitis C virus infection in Guinea-Bissau: a sexually transmitted genotype 2 with parenteral amplification? PLOS ONE. 2007;2(4):e372.

33. Abdool Karim SS, Tait DR. Hepatitis C virus infection in urban and rural Natal/KwaZulu. South African medical journal = Suid-Afrikaanse tydskrif vir geneeskunde. 1993;83(3):191-3.

34. Quesada P, Whitby D, Benavente Y, Miley W, Labo N, Chichareon S, et al. Hepatitis C virus seroprevalence in the general female population from 8 countries. Journal of clinical virology : the official publication of the Pan American Society for Clinical Virology. 2015;68:89-93.

35. Delaporte E, Thiers V, Dazza MC, Romeo R, Mlika-Cabanne N, Aptel I, et al. High level of hepatitis C endemicity in Gabon, equatorial Africa. TRANSACTIONS OF THE ROYAL SOCIETY OF TROPICAL MEDICINE AND HYGIENE. 1993;87(6):636-7.

36. Louis FJ, Maubert B, Le Hesran JY, Kemmegne J, Delaporte E, Louis JP. High prevalence of anti-hepatitis C virus antibodies in a Cameroon rural forest area. TRANSACTIONS OF THE ROYAL SOCIETY OF TROPICAL MEDICINE AND HYGIENE. 1994;88(1):53-4.

37. Ndong-Atome GR, Makuwa M, Ouwe-Missi-Oukem-Boyer O, Pybus OG, Branger M, Le Hello S, et al. High prevalence of hepatitis C virus infection and predominance of genotype 4 in rural Gabon. JOURNAL OF MEDICAL VIROLOGY. 2008;80(9):1581-7.

38. Njouom R, Pasquier C, Ayouba A, Gessain A, Froment A, Mfoupouendoun J, et al. High rate of hepatitis C virus infection and predominance of genotype 4 among elderly inhabitants of a remote village of the rain forest of South Cameroon. JOURNAL OF MEDICAL VIROLOGY. 2003;71(2):219-25.

39. Ayele W, Nokes DJ, Abebe A, Messele T, Dejene A, Enquselassie F, et al. Higher prevalence of anti-HCV antibodies among HIV-positive compared to HIV-negative inhabitants of Addis Ababa, Ethiopia. JOURNAL OF MEDICAL VIROLOGY. 2002;68(1):12-7.

40. Japhet M, Adewumi M, Olufisayo A. HIV, HBV AND HCV PREVALENCE, CO-INFECTIONS, RISK FACTORS AND AWARENESS AMONG STUDENTS IN A NIGERIAN UNIVERSITY. BMJ GLOBAL HEALTH. 2019;4:A59.

41. Laukmamm-Josten U, Ocheng D, Mwizarubi BK, Swai R, Nyamurekunge K. HIV, hepatitis B and C seroprevalence in truckstops and near by communities in Tanzania. Proceedings of the Ninth International Conference on AIDS/III. 1993.

42. Berhe N, Myrvang B, Gundersen SG. Intensity of Schistosoma mansoni, hepatitis B, age, and sex predict levels of hepatic periportal thickening/fibrosis (PPT/F): a large-scale community-based study in Ethiopia. The American journal of tropical medicine and hygiene. 2007;77(6):1079-86.

43. Kallestrup P, Zinyama R, Gomo E, Dickmeiss E, Platz P, Gerstoft J, et al. Low prevalence of hepatitis C virus antibodies in HIV-endemic area of Zimbabwe support sexual transmission as the major route of HIV transmission in Africa. AIDS. 2003;17(9):1400-2.

44. Kamdem SD, Konhawa F, Kuemkon EM, Meyo Kamguia L, Tchanana GK, Nche F, et al. Negative Association of Interleukin-33 Plasma Levels and Schistosomiasis Infection in a Site of Polyparasitism in Rural Cameroon. FRONTIERS IN IMMUNOLOGY. 2019;10:2827.

45. Njouom R, Caron M, Besson G, Ndong-Atome G-R, Makuwa M, Pouillot R, et al. Phylogeography, risk factors and genetic history of hepatitis C virus in Gabon, central Africa. PLOS ONE. 2012;7(8):e42002.

46. Ogefere HO, Moses-Otutu IM, Igiezeme CI. Potential risk factors and seroprevalence of hepatitis c virus infection among students in a tertiary institution in Southern Nigeria. Journal of Medicine and Biomedical Research. 2016;15(2):5-11.

47. Taye BD, Kassa T, Teshager L, Kedir R, Yeshanew AG, Aseffa A, et al. Prevalence and associated risk factors of Hepatitis B and C virus infections among mothers in Jimma, South West Ethiopia: a community-based study. Ethiop med j (Online). 2019;57(3):109-17.

48. Ramyil SC, Nimzing L, Lar N, Jonah PY, Dafam DD, Shik LP, et al. Prevalence and coinfection of human immunodeficiency virus, hepatitis B virus and hepatitis c virus in mabudi rural community of Plateau, State, Nigeria. SEXUALLY TRANSMITTED DISEASES. 2014;41:S144.

49. Mbopi-Keou F-X, Nkala IVM, Kalla GCM, Nguefack-Tsague G, Kamga HG, Noubom M, et al. [Prevalence and factors associated with HIV and viral hepatitis B and C in the city of Bafoussam in Cameroon]. Seroprevalence et facteurs associes au VIH et aux hepatites virales B et C dans la ville de Bafoussam au Cameroun. 2015;20:156.

50. Olalekan AW. Prevalence and risk factors for hepatitis B and C among sexually active undergraduates in southwestern Nigeria. ANNALS OF TROPICAL MEDICINE AND PUBLIC HEALTH. 2015;8(6):235-40.

51. Mencarini P, De Luca A, Antinori A, Maiuro G, Spedini G, Bailly C, et al. Prevalence of anti-HCV antibodies in Cameroon. TRANSACTIONS OF THE ROYAL SOCIETY OF TROPICAL MEDICINE AND HYGIENE. 1991;85(5):654-5.

52. Agbor VN, Tagny CT, Kenmegne J-B, Awazi B, Ngansop C, Mbanya D, et al. Prevalence of anti-hepatitis C antibodies and its co-infection with HIV in rural Cameroon. BMC RESEARCH NOTES. 2018;11(1):459.

53. Diallo MS, Sylla A, Sidibe K, Sylla BS, Trepo CR, Wild CP. Prevalence of exposure to aflatoxin and hepatitis B and C viruses in Guinea, West Africa. Natural toxins. 1995;3(1):6-9.

54. Pennap GR, Yakubu A, Oyige O, Forbi J. Prevalence of hepatitis B and C virus infection among people of a local community in Keffi, Nigeria. AFRICAN JOURNAL OF MICROBIOLOGY RESEARCH. 2010;4(4):274-8.

55. Malu AO, Achinge GI, Utoo PM, Kur JT, Obekpa SA. Prevalence of Hepatitis B Surface Antigen and Antibodies to Hepatitis C in the General Population of Benue State, Central Nigeria. The American journal of tropical medicine and hygiene. 2020;102(5):995-1000.

56. Mabayoje VO, Akinwusi PO, Opaleye O, Egbewale BE, Fagbami AH. Prevalence of hepatitis B surface antigen, hepatitis C and human immunodeficiency virus antibodies in a population of students of tertiary institution in Nigeria. Afr J Clin Exp Microbiol. 2010;11(2):68-74.

57. Okonkwo UC, Okpara H, Otu A, Ameh S, Ogarekpe Y, Osim H, et al. Prevalence of hepatitis B, hepatitis C and human immunodeficiency viruses, and evaluation of risk factors for transmission: Report of a population screening in Nigeria. SAMJ SOUTH AFRICAN MEDICAL JOURNAL. 2017;107(4):346-51.

58. Kowo MP, Goubau P, Ndam EC, Njoya O, Sasaki S, Seghers V, et al. Prevalence of hepatitis C virus and other blood-borne viruses in Pygmies and neighbouring Bantus in southern Cameroon. TRANSACTIONS OF THE ROYAL SOCIETY OF TROPICAL MEDICINE AND HYGIENE. 1995;89(5):484-6.

59. Jemilohun AC, Oyelade BO, Oiwoh SO. Prevalence of Hepatitis C virus antibody among undergraduates in Ogbomosho, South-Western Nigeria. Afr j infect dis (Online). 2014;8(2):40-3.

60. Omolade O, Adeyemi A. Prevalence of hepatitis C virus antibody among university students in Nigeria. JOURNAL OF VIRUS ERADICATION. 2018;4(4):228-9.

61. Ly KN, Kim AA, Drobeniuc J, Kodani M, Montgomery JM, Fields BS, et al. The Prevalence of Hepatitis C Virus Antibody in HIV-Negative Persons in Kenya, 2007. The American journal of tropical medicine and hygiene. 2018;98(6):1876-9.

62. Ntagirabiri R, Baransaka E, Ndayiragije A, Niyongabo T. Prevalence of hepatitis C virus in Burundi: A nationwide survey. Journal Africain d'Hepato-Gastroenterologie. 2014;8(1):25-8.

63. Zeller H, Rabarijaona L, RakotoAndrianarivelo M, Boisier P. Prevalence of hepatitis C virus infection in general population in Madagascar. BULLETIN DE LA SOCIETE DE PATHOLOGIE EXOTIQUE. 1997;90(1):3-5.

64. Ouedraogo A, Kabore M, Barry A, Coulibaly S, Ouattara D, Kargougou DW, et al. Prevalence of HIV, hepatitis B and hepatitis C infection among potential participants to experimental vaccines trials in a rural area of Burkina Faso. AMERICAN JOURNAL OF TROPICAL MEDICINE AND HYGIENE. 2018;99(4):345.

65. Iradukunda PG, Habyarimana T, Niyonzima FN, Uwitonze A-Y, Mpunga T. Risk factors associated with hepatitis B and C in rural population of Burera district, Rwanda. The Pan African medical journal. 2020;35:43.

66. Sentjens RE, Sisay Y, Vrielink H, Kebede D, Reesink HW. Risk factors for HCV, HTLV-I/II and HIV infection in various population subsets in Ethiopia. TRANSFUSION. 2000;40(10):80S-S.

67. Pepin J, Lavoie M, Pybus OG, Pouillot R, Foupouapouognigni Y, Rousset D, et al. Risk factors for hepatitis C virus transmission in colonial Cameroon. Clinical infectious diseases : an official publication of the Infectious Diseases Society of America. 2010;51(7):768-76.

68. Makuza JD, Liu CY, Ntihabose CK, Dushimiyimana D, Umuraza S, Nisingizwe MP, et al. Risk factors for viral hepatitis C infection in Rwanda: results from a nationwide screening program. BMC INFECTIOUS DISEASES. 2019;19(1):688.

69. Bile K, Mohamud O, Aden C, Isse A, Norder H, Nilsson L, et al. The risk for hepatitis A, B, and C at two institutions for children in Somalia with different socioeconomic conditions. The American journal of tropical medicine and hygiene. 1992;47(3):357-64.

70. Omer RE, Van't Veer P, Kadaru AM, Kampman E, el Khidir IM, Fedail SS, et al. The role of hepatitis B and hepatitis C viral infections in the incidence of hepatocellular carcinoma in Sudan. TRANSACTIONS OF THE ROYAL SOCIETY OF TROPICAL MEDICINE AND HYGIENE. 2001;95(5):487-91.

71. Makuza JD, Nisingizwe MP, Rwema JOT, Dushimiyimana D, Habimana DS, Umuraza S, et al. Role of unsafe medical practices and sexual behaviours in the hepatitis B and C syndemic and HIV co-infection in Rwanda: a cross-sectional study. BMJ OPEN. 2020;10(7):e036711.

72. Umutesi J, Liu CY, Penkunas MJ, Makuza JD, Ntihabose CK, Umuraza S, et al. Screening a nation for hepatitis C virus elimination: a cross-sectional study on prevalence of hepatitis C and associated risk factors in the Rwandan general population. BMJ OPEN. 2019;9(7):e029743.

73. Nansseu JR, Mbogning DM, Monamele GC, Tamoh SF, Gonsu HK, Kouanfack C, et al. Sero-epidemiology of human immunodeficiency virus, hepatitis B virus and hepatitis C virus: a cross-sectional survey in a rural setting of the West region of Cameroon. The Pan African medical journal. 2017;28:201.

74. Martinson FE, Weigle KA, Mushahwar IK, Weber DJ, Royce R, Lemon SM. Seroepidemiological survey of hepatitis B and C virus infections in Ghanaian children. JOURNAL OF MEDICAL VIROLOGY. 1996;48(3):278-83.

75. Alkali M, Okon KO, Umar MS, Sani MD, Shuaibu H, Babale RS, et al. SEROEPIDEMIOLOGY OF HBV ANTIGEN AND ANTI-HCV AMONG GENERAL POPULATION IN A RURAL LOCAL IN BAUCHI STATE, NIGERIA. West African journal of medicine. 2023(12 Suppl 1):S33-S4.

76. Jeremiah ZA, Tony-Enwin EO. Seroepidemiology of Transfusion Transmissible Viral Infection among University Fresh Students in Port Harcourt, Nigeria. HEPATITIS MONTHLY. 2009;9(4):276-81.

77. Stark K, Poggensee G, Hohne M, Bienzle U, Kiwelu I, Schreier E. Seroepidemiology of TT virus, GBC-C/HGV, and hepatitis viruses B, C, and E among women in a rural area of Tanzania. JOURNAL OF MEDICAL VIROLOGY. 2000;62(4):524-30.

78. Forbi J, Pennap G, Silas-Ndukuba C, Agabi Y, Agwale S. Serological markers and risk factors for hepatitis B and hepatitis C viruses among students in a Nigerian university. East African journal of public health. 2009;6(2):152-5.

79. Eke CB, Ogbodo SO, Ukoha OM, Muoneke VU, Ibekwe RC, Ikefuna AN. Seroprevalence and correlates of hepatitis c virus infection in secondary school children in Enugu, Nigeria. ANNALS OF MEDICAL AND HEALTH SCIENCES RESEARCH. 2016;6(3):156-61.

80. Basimane-Bisimwa P, Koyaweda GW, Ngaiganam E, Vickos U, Sibiro OAD, Yambiyo BM, et al. Seroprevalence and molecular characterization of viral hepatitis and HIV co-infection in the Central African Republic. medRxiv. 2023.

81. Ngatchu T, Stroffolini T, Rapicetta M, Chionne P, Lantum D, Chiaramonte M. Seroprevalence of anti-HCV in an urban child population: a pilot survey in a developing area, Cameroon. The Journal of tropical medicine and hygiene. 1992;95(1):57-61.

82. Muro FJ, Fiorillo SP, Sakasaka P, Odhiambo C, Reddy EA, Cunningham CK, et al. Seroprevalence of hepatitis B and C viruses among children in Kilimanjaro Region, Tanzania. JOURNAL OF THE PEDIATRIC INFECTIOUS DISEASES SOCIETY. 2013;2(4):320-6.

83. Basaras M, Santamaria A, Sarsa M, Gutierrez E, de Olano Y, Cisterna R. Seroprevalence of hepatitis B and C, and human immunodeficiency type 1 viruses in a rural population from the Republic of Equatorial Guinea. TRANSACTIONS OF THE ROYAL SOCIETY OF TROPICAL MEDICINE AND HYGIENE. 1999;93(3):250-2.

84. Abebe F, Seyoum B, Teklemariam Z, Oljira L, Tarekegne A, Bekele F, et al. Seroprevalence of Hepatitis B virus surface antigen, anti-hepatitis C virus antibody and their associated factors among mothers living in Harar, Eastern Ethiopia. Ethiop med j (Online). 2019;57(3):119-27.

85. Ramarokoto CE, Rakotomanana F, Ratsitorahina M, Raharimanga V, Razafindratsimandresy R, Randremanana R, et al. Seroprevalence of hepatitis C and associated risk factors in urban areas of Antananarivo, Madagascar. BMC INFECTIOUS DISEASES. 2008;8:25.

86. Kpossou AR, Kouwakanou B, Sokpon CNDM, Alassane KS, Bankole MM, Ahouada C, et al. [Seroprevalence of hepatitis C virus (HCV) antibodies and associated factors based on voluntary screening data collection in the general population in Benin in 2016]. Seroprevalence des anticorps anti-virus de l'hepatite C et facteurs associes, d'apres un depistage volontaire en population generale en 2016 au Benin. 2021;40:30.

87. Tess BH, Levin A, Brubaker G, Shao J, Drummond JE, Alter HJ, et al. Seroprevalence of hepatitis C virus in the general population of northwest Tanzania. The American journal of tropical medicine and hygiene. 2000;62(1):138-41.

88. Ejele OA, Nwauche CA, Erhabor O. Seroprevalence of hepatitis C virus in the Niger Delta of Nigeria. The Nigerian postgraduate medical journal. 2006;13(2):103-6.

89. Hodges M, Sanders E, Aitken C. Seroprevalence of hepatitis markers; HAV, HBV, HCV and HEV amongst primary school children in Freetown, Sierra Leone. West African journal of medicine. 1998;17(1):36-7.

90. Miller WC, Shao JF, Weaver DJ, Shimokura GH, Paul DA, Lallinger GJ. Seroprevalence of viral hepatitis in Tanzanian adults. Tropical medicine & international health : TM & IH. 1998;3(9):757-63.

91. Muhibi MA, Ifeanyichukwu MO, Olawuyi AO, Abulude AA, Adeyemo MO, Muhibi MO. Surveillance of anti-HCV antibody amongst in-school youth in a Nigeria university. African Journal of Clinical and Experimental Microbiology. 2019;20(1):49-53.

92. Frommel D, Tekle-Haimanot R, Berhe N, Aussel L, Verdier M, Preux PM, et al. A survey of antibodies to hepatitis C virus in Ethiopia. The American journal of tropical medicine and hygiene. 1993;49(4):435-9.

93. Kumalo A, Teklu T, Demisse T, Anjulo A. Undiagnosed Seroprevalence of Hepatitis B and C Virus Infections in the Community of Wolaita Zone, Southern Ethiopia. HEPATIC MEDICINE-EVIDENCE AND RESEARCH. 2022;14:111-22.

94. Forbi JC, Pietzsch J, Olaleye VO, Forbi TD, Pennap GR, Esona MD, et al. Urban-rural estimation of hepatitis C virus infection sero-prevalence in north Central Nigeria. East African journal of public health. 2010;7(4):367-8.
